# Supplementary material for: Real-time HIL validation of a lyapunov-guaranteed optimal FOPID controller for quadrotor UAV trajectory tracking
Source: Sci Rep. 2026 Aug 5;16:24167. doi: 10.1038/s41598-026-63836-x (PMC13443532; doi:10.1038/s41598-026-63836-x)
Supplement: Supplementary file 1 [file 41598_2026_63836_MOESM1_ESM.pdf]

Real-Time HIL Validation of a Lyapunov-guaranteed optimal FOPID controller for quadrotor trajectory tracking

Bassam Elbasueny[1], **Mohamed M. El-Sotouhy**[2], **Ehab Said**[1], **Ibrahim Elsherif**[1], **and Mohamed A. Kamel**[1,*]

[1]Department of Mechatronics Engineering, Military Technical College, Cairo, 11766, Egypt
[2]Power Electronics and Energy Conversion Dept., Electronics Research Institute (ERI), Cairo, 12622, Egypt
[*]mohamed.atef.kamel@mtc.edu.eg

ABSTRACT

This paper presents the real-time Hardware-in-the-Loop (HIL) validation of a robust 6-Degree-of-Freedom (6-DoF) Fractional-Order Proportional-Integral-Derivative (FOPID) control architecture, optimally tuned via the Artificial Gorilla Troops Optimizer (GTOA), for quadrotor Unmanned Aerial Vehicle (UAV) trajectory tracking. First, a comprehensive kinematic and dynamic model is developed, and the control architecture is designed by decoupling the UAV into fully-actuated and under-actuated subsystems through a cascaded inner-outer loop strategy. The global asymptotic stability is mathematically established via Lyapunov theory, and a dynamic Lyapunov penalty mechanism is further integrated into the GTOA objective function to enforce this stability condition across all candidate solutions throughout the optimization process. Subsequently, GTOA is applied to obtain the optimal gains that minimize the Integral of Time-Weighted Absolute Error (ITAE). Real-time HIL experiments are conducted on an industrial OPAL-RT OP4510 real-time target simulator, where the proposed GTOA-FOPID is evaluated against the African Vultures Optimization Algorithm (AVOA)-FOPID, alongside the baseline Particle Swarm Optimization (PSO)-tuned fractional-order and integer-order PID controllers. This comparative study evaluates the proposed architecture from two independent perspectives. First, the fractional-order operators are assessed against a PSO-tuned integer-order PID controller, isolating the contribution of the fractional calculus extension. Second, the GTOA is evaluated against AVOA and PSO, representing a modern metaheuristic and a standard baseline, respectively, across the identical FOPID architecture, isolating the contribution of the optimization strategy. Experimental results demonstrate the superiority of the fractional operators over the traditional integer-order PID controller. Furthermore, the proposed GTOA-FOPID achieves superior transient performance, precise trajectory tracking, and enhanced robustness under realistic hardware constraints and computational delays.

1 Introduction

In recent years, unmanned and autonomous systems have emerged as invaluable assets in modern technology, enabling complex operations without direct human intervention, particularly in hazardous, unstructured, or inaccessible environments. Autonomous vehicles are classified based on their operational domains into unmanned ground vehicles (UGVs), unmanned surface vehicles (USVs), unmanned underwater vehicles (UUVs), and unmanned aerial vehicles (UAVs)[1]. Among these types, UAVs have attracted attention from both academia and industry due to their extensive deployment across diverse civil and industrial applications, including infrastructure inspection, precise agricultural monitoring, payload delivery, and search-and-rescue operations[2,3].

Within many types of UAVs, quadrotors are widely used due to their stability, high maneuverability, redundancy, compactness, and low cost. The mechanical simplicity of the quadrotor—achieving six degrees-of-freedom (6-DoF) through the differential thrust of four fixed-pitch rotors—yields exceptional maneuverability and hovering capabilities[4]. Also, their ability to maintain steady flight at low velocities makes them particularly suitable for tasks that require precise positioning, stationary monitoring, or accurate payload deployment[5]. Moreover, the symmetric configuration of quadrotors ensures balanced aerodynamic forces, allowing for fast dynamic response and the execution of agile flight maneuvers[6]. However, from a control engineering perspective, quadrotors constitute highly coupled, underactuated, and nonlinear dynamic systems subject to aerodynamic disturbances and parametric uncertainties[7,8]. Consequently, synthesizing robust and computationally efficient flight control architectures remains a challenge in the field of autonomous systems.

In the literature, various control strategies have been employed for quadrotor UAVs such as Active Disturbance Rejection Control (ADRC)[9], Sliding Mode Control (SMC)[10,11], fuzzy logic control[12], Artificial Neural Network (ANN)[13], and backstepping[14]. Although these strategies offer robust performance, their real-time adoption is frequently limited by the high

computational overhead, accurate dynamic modeling requirements, and actuator chattering, which is especially associated with the SMC approaches[11]. Moreover, Model Predictive Control (MPC)[15] stands out as a highly effective optimization-based strategy. Recent advancements in MPC such as predictive functional control based on auxiliary variable optimization[16] and dynamic matrix control[17] have demonstrated the superiority of these methods in handling multi-variable constraints and executing direct online optimization. However, the real-time adoption of these advanced strategies is also limited by severe computational overhead. On the other hand, the classical Proportional-Integral-Derivative (PID) controller remains the standard in quadrotors flight controllers due to its structural simplicity and ease of implementation. Nevertheless, conventional PID controllers often struggle to maintain optimal transient and steady-state performance across the entire operational flight of highly nonlinear multi-rotor systems. To mitigate these limitations, the Fractional-Order PID (FOPID) controller is proposed as a compelling alternative.

In recent years, fractional calculus has evolved from a theoretical subject into a versatile mathematical tool with broadly practical applications[18,19]. Formulated upon fractional calculus, the FOPID extends the classical integer-order PID structure by introducing two additional degrees of freedom: the fractional integration order (λ) and the fractional differentiation order (μ), yielding the generalized $PI^{\lambda}D^{\mu}$ controller[20]. These extra parameters provide an iso-damping property and expanded frequency-domain tuning flexibility[21]. Consequently, FOPID controllers exhibit enhanced robustness against both parameter variations and external environmental disturbances compared to their integer-order counterparts[19]. With this configuration, FOPID allows for more flexible and accurate control, especially for systems with complex dynamics such as quadrotor UAVs[20].

Several studies have explored FOPID implementation for quadrotor platforms. In[22], FOPID is applied to 1-DoF vertical takeoff and landing (VTOL) system, while Genetic Algorithm (GA) is applied to find the optimal parameters; however only simulation results are presented. In[23], an FOPID is applied for a Twin Rotor MIMO System (TRMS) and testing it on physical laboratory hardware. Three different metaheuristic algorithms are applied to obtain the optimal tuning parameters: GA, the Particle Swarm Optimization (PSO), and the Nelder-Mead (NM) algorithm. Nevertheless, despite this work explicitly acknowledges that the twin-rotor system is a highly nonlinear, cross-coupled system, the controller design is based on linear model approximation. In[24], FOPID is applied for set point tracking and sinusoidal disturbance rejection in quadrotors. In this work, controller parameters are tuned manually, results in high complexity. Moreover, simulation results are presented. Recent efforts have integrated hybrid strategies, such as the Simulated Annealing (SA)-PSO algorithm[25], or focused on specialized scenarios like propeller failure[26], where the Oustaloup approximation is utilized for real-time testing on an educational prototype of the Tello UAV. In[27,28], Bonobo Optimization (BO) is applied for the optimal tuning of the FOPID controller. Simulation results are mentioned while it can be noticed that FOPID tuning demands a high number of iterations and population size, increasing computational burden.

As can be noticed from the existing literature, despite its theoretical advantages, the practical implementation of FOPID controllers is severely constrained by the complexity of its tuning process as two more parameters (μ and λ) are added to the controller. Consequently, metaheuristic optimization algorithms have been widely adopted to search the high-dimensional non-convex parameter space. Traditional algorithms, such as GA[29] and PSO[20], often exhibit premature convergence to local optima and suffer from poor exploration-exploitation balance when tasked with complex multi-objective tuning problems. Other techniques, such as Differential Evolution (DE), offer recognized robustness within continuous optimization landscapes[30]. However, the efficacy of DE is fundamentally reliant upon the precise calibration of its mutation strategies and control variables, rendering the algorithm susceptible to instability during the search process. Likewise, while the Artificial Bee Colony (ABC) framework provides commendable global exploration capabilities[31], its inherently slow convergence rate often imposes prohibitive computational costs when applied to highly complex objective functions. Subsequent developments in nature-inspired heuristics, notably the Grey Wolf Optimizer (GWO) and the Whale Optimization Algorithm (WOA), were introduced to enhance the equilibrium between global exploration and local exploitation[32]. Nevertheless, and despite their advantages, these methods remain susceptible to entrapment in local optima, especially with the case of nonlinear objective functions. To overcome the limitations of these approaches, recent advancements in nature-inspired heuristics, specifically the Artificial Gorilla Troops Optimizer (GTOA)[33] and the African Vultures Optimization Algorithm (AVOA)[34], demonstrate superior global search capabilities and faster convergence rates, presenting a viable solution for optimal FOPID synthesis.

Based on the aforementioned review of the literature, the main technical challenges regarding quadrotor UAV control can be summarized as follows:

1. While advanced nonlinear control strategies offer robust performance, their real-time validation is frequently limited by the high computational effort;

2. Despite the simplicity and applicability of the traditional PID controllers, they often struggle to maintain optimal transient and steady-state performance; and

3. Although the advantages of the FOPID controller, its practical implementation is constrained by the complexity of tuning its parameters. Consequently, the majority of existing studies consider only the numerical simulations, and have not been

validated in real-time testings.

To address these gaps, this work extends the authors' preliminary research[35] by presenting a comprehensive, theoretically grounded, and hardware-validated optimal FOPID control framework for quadrotor trajectory tracking in the presence of model decoupling, nonlinearities, and external disturbances. The control architecture employs a decoupling strategy that divides the UAV dynamics into fully-actuated and under-actuated subsystems, further structured into a cascaded inner-outer loop configuration. To achieve optimal performance, GTOA is applied to find the optimal parameters of the control architecture, with the objective of minimizing the integral time-weighted absolute error (ITAE) of the UAV pose. To guarantee the stability of the fractional-order closed-loop system, a two-layer stability framework is established. At the analytical layer, Lyapunov theory is employed to derive the stability condition and prove the asymptotic convergence of the tracking error dynamics for the nonlinear time-varying quadrotor states. At the computational layer, the derived Lyapunov condition is embedded as a hard penalty constraint within the GTOA objective function, ensuring that every candidate solution generated during the optimization process is guaranteed to satisfy the asymptotic stability requirement.

As compared to the existing studies in the literature, the main contributions of this work can be outlined as follows:

1. Developing a comprehensive 6-DoF cascaded control architecture based on FOPID, explicitly accounting for UAV nonlinearities, and model coupling;
2. Applying the advanced metaheuristic algorithm, the GTOA, to synthesize the optimal FOPID parameters by minimizing the ITAE of the UAV state tracking errors, supported by a robust comparative analysis against AVOA-FOPID, PSO-FOPID and PSO-PID;
3. Establishing a two-layer stability framework in which Lyapunov theory analytically derives the asymptotic stability condition, while a dynamic Lyapunov penalty mechanism is embedded within the GTOA objective function to computationally enforce this condition across all candidate solutions throughout the optimization process, thereby guaranteeing the global asymptotic stability of the closed-loop system; and
4. Performing real-time physical validation of the proposed fractional-order control architecture on an industrial OPAL-RT OP4510 HIL platform.

The remainder of the paper is organized as follows: Section 2 formulates the complete kinematic and dynamic mathematical model of the 6-DoF quadrotor UAV. Section 3 details the proposed control architecture, including the system decoupling strategy, the formulation of the FOPID controllers, the stability proof utilizing Lyapunov's criteria, and the implementation of the optimization algorithms. Section 4 provides the physical validation of the proposed framework via real-time HIL experiments. Finally, conclusions are outlined in Section 5.

2 Quadrotor Model

This section presents the complete kinematic and dynamic model of the quadrotor UAV. The vehicle's configuration is defined using two coordinate systems: an earth-fixed inertial frame ($\mathbb{E}$) and a body-fixed frame ($\mathbb{B}$) originating at the quadrotor's center of mass. The transformation mapping between these frames is parameterized via standard Euler angles—roll (ϕ), pitch (θ), and yaw (ψ)[36,37].

A quadrotor operates as a highly coupled, 6-DoF underactuated rigid body. As illustrated in Figure 1, the UAV features a

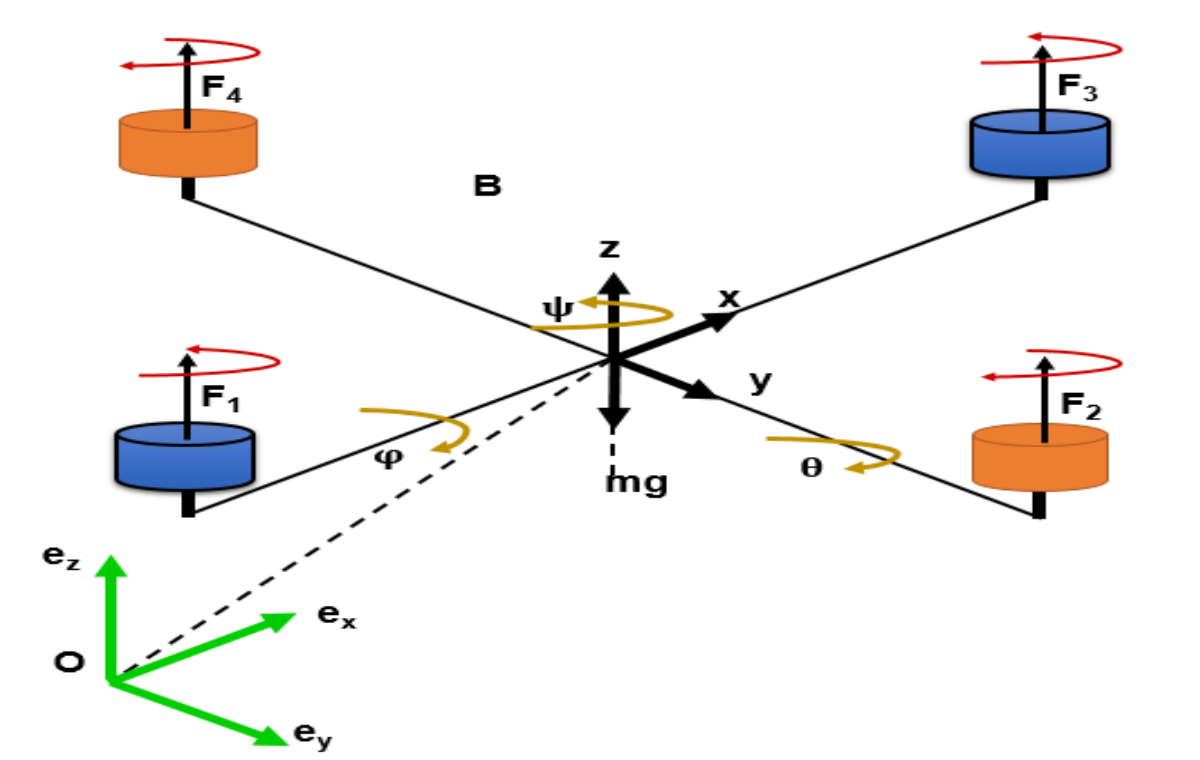

Figure 1. Quadrotor aircraft motion.

symmetric, cross-configured frame housing four independent rotors, grouped into two opposing pairs: rotors (1, 3) and rotors (2, 4). The main concept of flight control relies on modulating the angular velocities of these four rotors to handle the net aerodynamic thrust and the resultant torques. Vertical translation (altitude) is governed by synchronously adjusting all four rotor speeds. Lateral and longitudinal translations are coupled with the roll and pitch attitude dynamics, respectively, which are induced by establishing differential thrust across opposing rotor pairs. Finally, directional heading (yaw control) is managed by exploiting the aerodynamic drag imbalance, achieved by varying the reactive counter-torques between the clockwise and counterclockwise rotating rotor pairs[38].

To facilitate real-time control synthesis, the following standard physical assumptions are established[38]:

Assumption 1. *The quadrotor possesses a perfect symmetrical physical structure with its center of mass aligning with the geometric center, and its frame is completely rigid.*

Assumption 2. *The propeller blades exhibit negligible deformation across all flight regimes.*

Assumption 3. *The aerodynamic thrust and drag forces produced by each individual rotor are proportional to the square of their respective angular velocities.*

Let the absolute position and translational velocity of the quadrotor's center of mass in the inertial frame $\mathbb{E}$ be denoted by the vectors $X = [x, y, z]^T$ and $V = [u, v, w]^T$, respectively. Similarly, the vehicle's attitude and angular velocity vectors, mapped in the body frame $\mathbb{B}$, are defined as $\Theta = [\phi, \theta, \psi]^T$ and $\Omega = [p, q, r]^T$. To prevent kinematic singularities, the operational envelope of the Euler angles is bounded as follows:

$$-\pi/2 < \phi < \pi/2,\ -\pi/2 < \theta < \pi/2,\ -\pi < \psi < \pi$$

The translational and rotational dynamics of the rigid body are derived using the standard Newton-Euler formulation[39]:

$$\begin{bmatrix} mI_{3\times 3} & 0 \\ 0 & I \end{bmatrix} \begin{bmatrix} \dot{V^B} \\ \dot{\Omega} \end{bmatrix} + \begin{bmatrix} \Omega \times mV^B \\ \Omega \times I\Omega \end{bmatrix} = \begin{bmatrix} F \\ \tau \end{bmatrix} \tag{1}$$

where m represents the UAV mass, and $I \in \mathbb{R}^{3\times 3}$ is the diagonal inertia tensor defined by the principal mass moments of inertia I_{xx}, I_{yy}, and I_{zz}. V^B is the translational velocity vector relative to the body frame, while F and τ denote the aerodynamic thrust and torques generated by the propulsion system, respectively.

To map the dynamic states from the body frame $\mathbb{B}$ to the inertial frame $\mathbb{E}$, the directional cosine matrix (rotation matrix) R is utilized:

$$R = \begin{bmatrix} C_\psi C_\theta & -S_\psi C_\phi + C_\psi S_\theta S_\phi & S_\psi S_\phi + C_\psi S_\theta C_\phi \\ S_\psi C_\theta & C_\psi C_\phi + S_\psi S_\theta S_\phi & -C_\psi S_\phi + S_\psi S_\theta C_\phi \\ -S_\theta & C_\theta S_\phi & C_\phi C_\theta \end{bmatrix} \tag{2}$$

where C_i and S_i correspond to $\cos(i)$ and $\sin(i)$, respectively. Consequently, the comprehensive nonlinear 6-DOF dynamic equations governing the quadrotor's flight while omitting the drag forces are formulated as follows[40]:

$$\ddot{x} = \frac{U_1}{m}(\sin\phi \sin\psi + \cos\phi \cos\psi \sin\theta) \tag{3}$$

$$\ddot{y} = \frac{U_1}{m}(\cos\phi \sin\psi \sin\theta + \cos\psi \sin\phi) \tag{4}$$

$$\ddot{z} = -g + \left(\frac{U_1}{m}(\cos\phi \cos\theta) + K_{dz}\dot{z}\right) \tag{5}$$

$$\ddot{\phi} = \frac{l}{I_{xx}}U_2 - \frac{J_r}{I_{xx}}\dot{\theta}\Omega_r + \frac{I_{yy}}{I_{xx}}\dot{\psi}\dot{\theta} - \frac{I_{zz}}{I_{xx}}\dot{\theta}\dot{\psi} \tag{6}$$

$$\ddot{\theta} = \frac{l}{I_{yy}}U_3 - \frac{J_r}{I_{yy}}\dot{\phi}\Omega_r + \frac{I_{zz}}{I_{yy}}\dot{\phi}\dot{\psi} - \frac{I_{xx}}{I_{yy}}\dot{\psi}\dot{\phi} \tag{7}$$

$$\ddot{\psi} = \frac{1}{I_{zz}}U_4 + \frac{I_{xx}}{I_{zz}}\dot{\theta}\dot{\phi} - \frac{I_{yy}}{I_{zz}}\dot{\phi}\dot{\theta} \tag{8}$$

where $U_i, i = 1, \ldots, 4$ represent the virtual control inputs mapping to the total thrust, roll, pitch, and yaw moments, respectively. g is the gravitational acceleration, J_r indicates the rotational inertia of a single rotor, Ω_r is the angular velocity of the propeller,

and l specifies distance between the quadrotor center of gravity and the motor.

The control inputs U_i can be calculated as follows:

$$U_1 = F_1 + F_2 + F_3 + F_4 \tag{9}$$
$$U_2 = l(F_3 - F_4) \tag{10}$$
$$U_3 = l(F_1 - F_2) \tag{11}$$
$$U_4 = K_{yaw}(F_1 + F_2 - F_3 - F_4) \tag{12}$$

where $F_i, i = 1, \ldots, 4$ is the propellers thrust produced by the ith propeller, and K_{yaw} is a constant related to a relationship between the thrust of the propeller with the yaw moment. Considering the UAV's motors as a first order system, the force of the ith motor is[41]:

$$F_i = K_T \frac{\omega_m}{s + \omega_m} u_i \tag{13}$$

where K_T is the steady-state thrust gain, ω_m represents the motor bandwidth, and u_i is the applied Pulse Width Modulation (PWM) command signal for the ith motor. Finally, the rotor speed Ω_r is defined as:

$$\Omega_r = -\Omega_1 + \Omega_2 - \Omega_3 + \Omega_4 \tag{14}$$

where Ω_i indicates the angular velocity of the ith individual propeller.

3 Controller Design and Stability Analysis

This section outlines the design and mathematical formulation of the proposed optimal quadrotor UAV trajectory tracking control architecture. Given the severe nonlinearities and aerodynamic coupling inherent to quadrotor UAVs, an FOPID framework is deployed. First, 6-DoF cascaded control scheme governing the UAV dynamics is formulated. Then, a two-layer stability framework is established: the global asymptotic stability of the closed-loop system is analytically proven via Lyapunov theory, followed by the formulation of a dynamic Lyapunov penalty mechanism that integrated into the objective function to enforce the stability across all candidate solutions throughout the optimization process. Finally, the optimal tuning problem is defined by specifying the objective function and the multi-dimensional parameters, where the GTOA is utilized to find the optimal values of the FOPID gains by minimizing the ITAE of the UAV state tracking errors.

3.1 Fractional-Order PID Control Architecture

The FOPID controller, denoted $PI^\lambda D^\mu$, extends the classical integer-order PID by introducing two additional degrees of freedom: the fractional integration order λ and the fractional differentiation order μ. The fractional operators are defined using the Caputo derivative, adopted for its compatibility with integer-order initial conditions[42]. The resulting time-domain control effort is:

$$u(t) = K_p\, e(t) + K_i\, {}_0^C D_t^{-\lambda} e(t) + K_d\, {}_0^C D_t^{\mu} e(t) \tag{15}$$

where K_p, K_i, and K_d are the proportional, integral, and derivative gains, respectively. The expanded five-dimensional parameter space $\{K_p, K_i, K_d, \lambda, \mu\}$ grants an iso-damping property that ensures enhanced robustness against parameter variations and aerodynamic disturbances compared to integer-order PID controllers[21]. However, analytically determining the optimal configuration for this five-dimensional space is highly complex, especially for the real-time deployment. Therefore, it is necessary to utilize a modern metaheuristic technique such as the GTOA, as detailed in Subsection 3.3.

The quadrotor is an underactuated nonlinear system, as six-DoF are controlled by only four independent physical inputs (U_1, U_2, U_3, U_4). To achieve precise trajectory tracking in three-dimensional space, a cascaded control architecture is adopted. This structure divides the flight dynamics into fully-actuated and under-actuated subsystems. The overall control system architecture is illustrated in Figure 2.

For any given state $k \in \{x, y, z, \phi, \theta, \psi\}$, the instantaneous tracking error is defined as $e_k(t) = k_r(t) - k(t)$, where the subscript r denotes the desired reference trajectory. A dedicated FOPID controller is assigned to each of the six state variables, producing a virtual control effort $u_k(t)$:

$$u_k(t) = K_{p,k} e_k(t) + K_{i,k}\, {}_0^C D_t^{-\lambda_k} e_k(t) + K_{d,k}\, {}_0 D_t^{\mu_k} e_k(t) \tag{16}$$

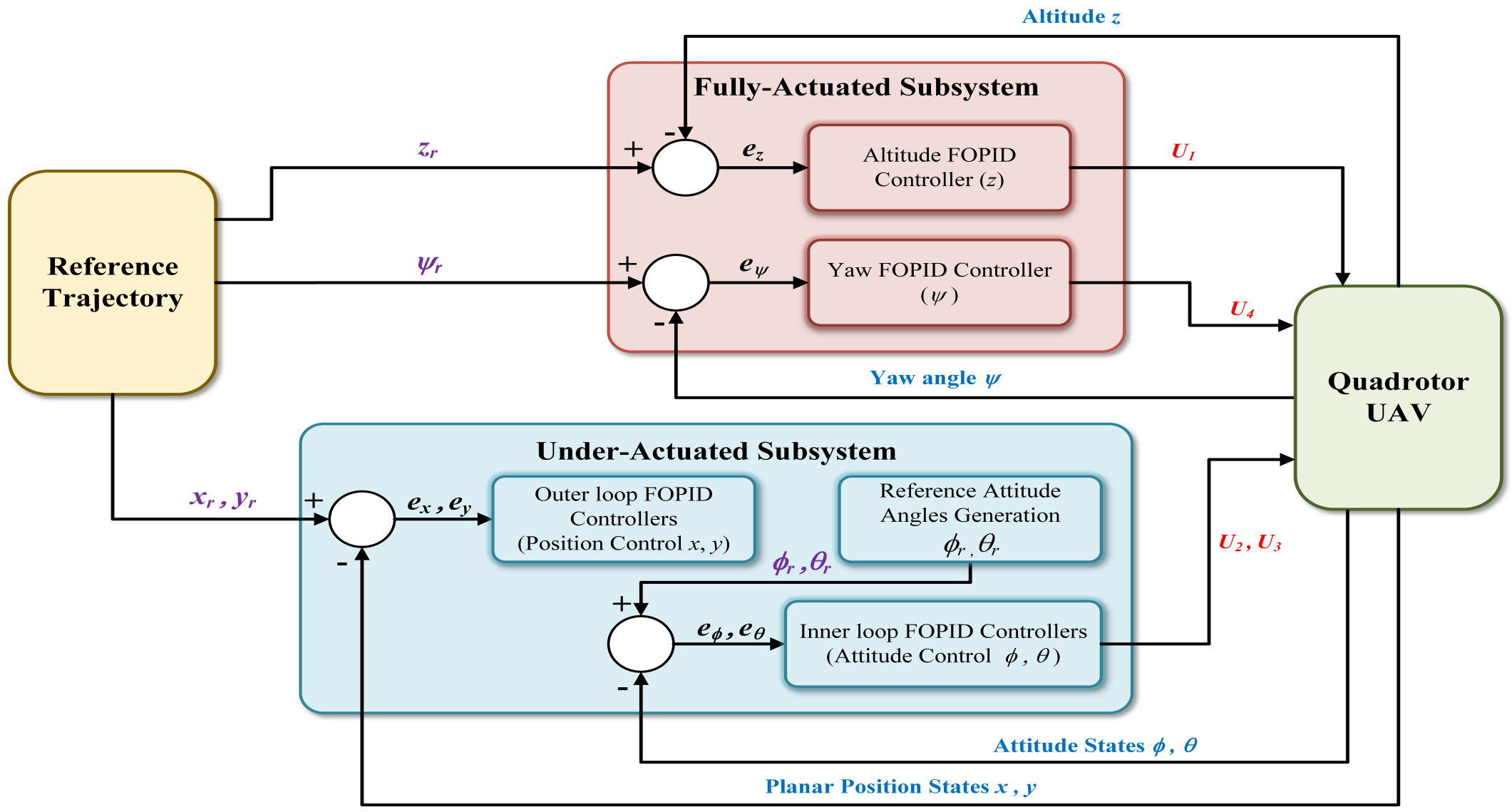

Figure 2. FOPID control architecture block diagram.

3.1.1 *Fully–Actuated Subsystem*

It is responsible for directly governing the altitude z and the yaw heading angle ψ, as mentioned in Eqs. 5 and 8, respectively. Therefore, the primary objective of this subsystem is to minimize the trajectory tracking errors in altitude and yaw, denoted by $e_z(t)$ and $e_\psi(t)$, respectively. Accordingly, the FOPID controllers designed for this subsystem must mathematically guarantee the following asymptotic convergence performance:

$$\lim_{t\to\infty} \|e_z(t)\| = \lim_{t\to\infty} \|z_r(t) - z(t)\| = 0 \tag{17}$$

$$\lim_{t\to\infty} \left\|e_\psi(t)\right\| = \lim_{t\to\infty} \|\psi_r(t) - \psi(t)\| = 0 \tag{18}$$

where $z_r(t)$ and $\psi_r(t)$ represent the desired reference altitude and yaw angle, respectively. To achieve these asymptotic conditions, the altitude and yaw FOPID controllers are formulated to generate the virtual control efforts $u_z(t)$ and $u_\psi(t)$ as follows:

$$u_z(t) = K_{p,z} e_z(t) + K_{i,z}\, {}_0^C D_t^{-\lambda_z} e_z(t) + K_{d,z}\, {}_0^C D_t^{\mu_z} e_z(t) \tag{19}$$

$$u_\psi(t) = K_{p,\psi} e_\psi(t) + K_{i,\psi}\, {}_0^C D_t^{-\lambda_\psi} e_\psi(t) + K_{d,\psi}\, {}_0^C D_t^{\mu_\psi} e_\psi(t) \tag{20}$$

Finally, to apply these control laws to the physical quadrotor plant while compensating for the nonlinear gravitational pull and the vehicle's moment of inertia, the virtual efforts are mapped to the total physical thrust (U_1) and the directional yaw torque (U_4) as follows:

$$U_1(t) = \frac{m}{\cos\phi\cos\theta}\left(g + u_z(t)\right) \tag{21}$$

$$U_4(t) = I_{zz}\left(u_\psi(t) - \frac{I_{xx} - I_{yy}}{I_{zz}}\dot{\theta}\dot{\phi}\right) \tag{22}$$

By incorporating the $\dot{\theta}\dot{\phi}$ directly into the U_4 mapping, the controller actively neutralizes the highly coupled nonlinear inertial dynamics, leaving the fractional integral action to handle unmodeled external aerodynamics.

3.1.2 *Under–Actuated Subsystem*

Because the quadrotor lacks direct physical actuators for horizontal translation, the lateral and longitudinal motions (x, y) are under-actuated and heavily coupled with the vehicle's attitude. To stabilize this subsystem, a cascaded architecture consisting of an outer position loop and an inner attitude loop is utilized[11].

Outer Loop (Planar Motion): This loop governs the trajectory of the quadrotor in the xy-plane. The primary objective is

to minimize the tracking errors, $e_x(t)$ and $e_y(t)$, satisfying the following asymptotic conditions:

$$\lim_{t\to\infty} \|e_x(t)\| = \lim_{t\to\infty} \|x_r(t) - x(t)\| = 0 \tag{23}$$

$$\lim_{t\to\infty} \left\|e_y(t)\right\| = \lim_{t\to\infty} \|y_r(t) - y(t)\| = 0 \tag{24}$$

where $x_r(t)$ and $y_r(t)$ represent the reference positions. The outer loop FOPID controllers process these errors to generate the necessary virtual control efforts $u_x(t)$ and $u_y(t)$:

$$u_x(t) = K_{p,x} e_x(t) + K_{i,x}\,{}_0^C D_t^{-\lambda_x} e_x(t) + K_{d,x}\,{}_0^C D_t^{\mu_x} e_x(t) \tag{25}$$

$$u_y(t) = K_{p,y} e_y(t) + K_{i,y}\,{}_0^C D_t^{-\lambda_y} e_y(t) + K_{d,y}\,{}_0^C D_t^{\mu_y} e_y(t) \tag{26}$$

As the horizontal acceleration is achieved solely by tilting the thrust vector, these virtual control signals are mapped using the system's dynamic model to generate the desired reference roll ϕ_r and pitch θ_r angles that will be the inputs to the inner loop as follows:

$$\phi_r(t) = \arcsin\left(\frac{m}{U_1(t)}\left(u_x(t)\sin\psi_r(t) - u_y(t)\cos\psi_r(t)\right)\right) \tag{27}$$

$$\theta_r(t) = \arcsin\left(\frac{m}{U_1(t)\cos\phi_r(t)}\left(u_x(t)\cos\psi_r(t) + u_y(t)\sin\psi_r(t)\right)\right) \tag{28}$$

Inner Loop (Attitude Control): This loop ensures rapid and stable tracking of the reference angles generated by the outer loop. The objective is to minimize the angular tracking errors, $e_\phi(t)$ and $e_\theta(t)$, such that:

$$\lim_{t\to\infty} \left\|e_\phi(t)\right\| = \lim_{t\to\infty} \|\phi_r(t) - \phi(t)\| = 0 \tag{29}$$

$$\lim_{t\to\infty} \|e_\theta(t)\| = \lim_{t\to\infty} \|\theta_r(t) - \theta(t)\| = 0 \tag{30}$$

The inner loop FOPID controllers calculate the following virtual rotational control efforts $u_\phi(t)$ and $u_\theta(t)$:

$$u_\phi(t) = K_{p,\phi} e_\phi(t) + K_{i,\phi}\,{}_0^C D_t^{-\lambda_\phi} e_\phi(t) + K_{d,\phi}\,{}_0^C D_t^{\mu_\phi} e_\phi(t) \tag{31}$$

$$u_\theta(t) = K_{p,\theta} e_\theta(t) + K_{i,\theta}\,{}_0^C D_t^{-\lambda_\theta} e_\theta(t) + K_{d,\theta}\,{}_0^C D_t^{\mu_\theta} e_\theta(t) \tag{32}$$

Finally, these virtual efforts are mapped to the physical roll and pitch torques U_2 and U_3, respectively as follows:

$$U_2(t) = \frac{I_{xx}}{l}\left(u_\phi(t) + \frac{J_r}{I_{xx}}\dot{\theta}\Omega_r - \frac{I_{yy} - I_{zz}}{I_{xx}}\dot{\psi}\dot{\theta}\right) \tag{33}$$

$$U_3(t) = \frac{I_{yy}}{l}\left(u_\theta(t) - \frac{J_r}{I_{yy}}\dot{\phi}\Omega_r - \frac{I_{zz} - I_{xx}}{I_{yy}}\dot{\phi}\dot{\psi}\right) \tag{34}$$

3.2 Lyapunov-Based Stability Analysis

To evaluate the stability of the physical quadrotor under the proposed FOPID architecture, let the highly coupled UAV dynamic model described by Eqs. (1) to (6) be expressed in the standard nonlinear control-affine form:

$$\ddot{X} = f(X, \dot{X}) + g(X)U \tag{35}$$

where $X = [x, y, z, \phi, \theta, \psi]^T \in \mathbb{R}^6$ represents the generalized state vector, $U = [U_1, U_2, U_3, U_4]^\top \in \mathbb{R}^4$ is the physical control input vector, $f : \mathbb{R}^6 \times \mathbb{R}^6 \to \mathbb{R}^6$ denotes the drift vector field encapsulating the aerodynamic and Coriolis nonlinearities, and $g : \mathbb{R}^6 \to \mathbb{R}^{6\times 4}$ is the control allocation matrix. The primary control objective is to ensure the state $X(t)$ asymptotically tracks a time-varying reference trajectory $X_r(t)$.

Assumption 4. *The desired user-defined reference trajectory vector $X_r(t)$ is physically realizable and sufficiently smooth. Specifically, $X_r(t)$ and its time derivatives up to the second order are continuous and strictly bounded such that:*

$$\|X_r(t)\| \le \rho_0, \ \ \|\dot{X}_r(t)\| \le \rho_1, \ \ \|\ddot{X}_r(t)\| \le \rho_2$$

where ρ_0, ρ_1, and ρ_2 are known positive constants.

To establish the stability of the nonlinear error dynamics, the tracking error vector is defined as $E(t) = X_r(t) - X(t)$. Applying the principles of exact feedback linearization through the inverse dynamics of the quadrotor, the nonlinearities in (35) are locally decoupled, yielding the closed-loop tracking error dynamics for each decoupled axis $k \in \{x, y, z, \phi, \theta, \psi\}$ as:

$$\ddot{e}_k(t) = -u_k(t) \tag{36}$$

where $u_k(t)$ is the virtual FOPID control effort. Substituting $u_k(t)$ into (36) yields:

$$\ddot{e}_k(t) + K_{p,k}e_k(t) + K_{i,k}\,{}_0^C D_t^{-\lambda_k} e_k(t) + K_{d,k}\,{}_0^C D_t^{\mu_k} e_k(t) = 0 \tag{37}$$

Theorem 1. *(Lyapunov Asymptotic Stability). For the nonlinear quadrotor UAV governed by the proposed FOPID control architecture, the closed-loop tracking error $E(t)$ converges asymptotically to zero ($\lim_{t\to\infty} \|E(t)\| = 0$) provided that the fractional tuning parameters Φ_k belong to the stabilizing subset $\mathbb{S}_{stable}$ that ensures the continuous dissipation of the Lyapunov energy function, i.e., $\dot{V}_k \leq 0$ holds for all $t \geq 0$.*

Proof. To mathematically prove the asymptotic convergence of the error dynamics, let us define the following positive-definite Lyapunov candidate function $V(e_k, \dot{e}_k)$ representing the generalized energy of the tracking error for axis k:

$$V_k = \frac{1}{2}\dot{e}_k^2(t) + \frac{1}{2}K_{p,k}e_k^2(t) \tag{38}$$

where $K_{p,k} > 0$ is the proportional gain. Clearly, $V_k > 0$ for all $e_k \neq 0$ and $\dot{e}_k \neq 0$, satisfying the foundational requirement of Lyapunov's direct method. Taking the first-order time derivative of the Lyapunov candidate function yields:

$$\dot{V}_k = \dot{e}_k(t)\ddot{e}_k(t) + K_{p,k}e_k(t)\dot{e}_k(t) \tag{39}$$

Substituting the nonlinear error dynamics from Eq. (37) into $\ddot{e}_k(t)$ gives:

$$\dot{V}_k = \dot{e}_k(t)\Big[-K_{p,k}e_k(t) - K_{i,k}\,{}_0^C D_t^{-\lambda_k} e_k(t) - K_{d,k}\,{}_0^C D_t^{\mu_k} e_k(t)\Big] + K_{p,k}e_k(t)\dot{e}_k(t) \tag{40}$$

The proportional energy terms, $-\dot{e}_k(t)K_{p,k}e_k(t)$ and $K_{p,k}e_k(t)\dot{e}_k(t)$, algebraically cancel out. This reduces the time derivative of the Lyapunov function to the fractional energy dissipation terms:

$$\dot{V}_k = -\dot{e}_k(t)\left[K_{i,k}\,{}_0^C D_t^{-\lambda_k} e_k(t) + K_{d,k}\,{}_0^C D_t^{\mu_k} e_k(t)\right] \tag{41}$$

According to Lyapunov's theorem for asymptotic stability, the system is strictly stable if $\dot{V}_k \leq 0$. Due to the memory characteristics and non-local properties of the fractional integration λ and differentiation μ, ensuring $\dot{V}_k < 0$ analytically across all flight envelopes is highly prohibitive.

Consequently, Eq. (41) establishes the necessary and sufficient stability condition: the system achieves global asymptotic stability if and only if the fractional dissipation terms satisfy $\dot{e}_k(t)\left[K_{i,k}\,{}_0^C D_t^{-\lambda_k} e_k(t) + K_{d,k}\,{}_0^C D_t^{\mu_k} e_k(t)\right] > 0$ for all $t \geq 0$. While analytically enforcing this condition across all flight envelopes and fractional parameter combinations is highly prohibitive due to the non-local memory properties of the fractional operators, this condition is precisely what is derived here and subsequently enforced computationally in Section 3.3 through the Lyapunov-constrained objective function. This two-layer approach — analytical derivation in Theorem 1 followed by computational enforcement in Corollary 1 — constitutes the complete stability guarantee of the proposed framework. □

Lemma 1. *(Boundedness of Physical Control Inputs). Under Assumption 4, and provided that the Lyapunov energy dissipation condition ($\dot{V}_k \leq 0$) is satisfied as established in Theorem 1, the physical control inputs (U_1, U_2, U_3, U_4) remain strictly bounded for all $t \geq 0$.*

Proof. By satisfying the Lyapunov stability condition formulated in Eq. (41), the generalized energy of the tracking error dissipates over time ($\dot{V}_k \leq 0$). Consequently, the tracking errors $e_k(t)$ and their time-derivatives $\dot{e}_k(t)$ are strictly bounded.

Since the instantaneous physical state is defined as $X(t) = X_r(t) - E(t)$, and the user-defined reference trajectory $X_r(t)$ is bounded per Assumption 4, it follows directly that all physical system states (e.g., ϕ, θ, ψ) and their respective angular rates ($\dot{\phi}, \dot{\theta}, \dot{\psi}$) remain bounded throughout the flight envelope.

The physical control inputs (U_1, U_2, U_3, U_4) are continuous algebraic mappings of the bounded virtual fractional efforts $u_k(t)$ and these bounded physical states. Because the operational flight envelope naturally restricts the quadrotor from reaching pitch/roll singularities (i.e., $\cos\phi \cos\theta \neq 0$), the control allocation matrix contains no infinite discontinuities. Therefore, there exists a positive finite constant U_{max} such that $\|U_i(t)\| \leq U_{max}$ for all $i \in \{1, 2, 3, 4\}$, mathematically ensuring the actuators are protected from infinite command saturation. □

Remark 1. *(Practical Robustness). While Theorem 1 provides the mathematical guarantee for nominal tracking convergence, real-world implementation involves unmodeled actuator saturation, discrete-time sampling delays, and communication latency. The true advantage of the FOPID controller lies in its iso-damping phase margin, which maintains the negative definiteness of $\dot{V}_k$ even when the drift vector $f(X,\dot{X})$ is subjected to these unmodeled physical perturbations. This hardware-level robustness is experimentally validated in Section 5.*

3.3 Optimal Calculation of Controllers' Gains

To achieve the asymptotic tracking performance proven in the Lyapunov stability analysis, the parameters of the six cascaded FOPID controllers must be optimally tuned. For any given controlled state $k \in \{x, y, z, \phi, \theta, \psi\}$, the fractional-order controller is governed by five design variables: the proportional, integral, and derivative gains, alongside the fractional integration and differentiation orders.

Let the vector of design variables Φ_k for the kth axis be defined as:

$$\Phi_k = [K_{p,k}, K_{i,k}, K_{d,k}, \lambda_k, \mu_k]^T, \quad \forall k \in \{x, y, z, \phi, \theta, \psi\}.$$

To navigate this highly complex parameter space and find the optimal parameter set, denoted as Φ_k^*, the GTOA is deployed. The optimization objective is to minimize the Integral of Time-weighted Absolute Error (ITAE), which is specifically selected because the time-weighting factor heavily penalizes long-duration transient responses and steady-state errors, thereby ensuring rapid convergence, minimal overshoot, and precise trajectory tracking.

For each individual controller, the optimization problem solved by the GTOA is formulated as:

$$\min_{\Phi_k} J_k(\Phi_k) = \int_0^\infty t|e_k(t)|dt \tag{42}$$

subject to the inequality constraints that define the multi-dimensional search space:

$$K_{p,min} \leq K_{p,k} \leq K_{p,max} \tag{43}$$
$$K_{i,min} \leq K_{i,k} \leq K_{i,max} \tag{44}$$
$$K_{d,min} \leq K_{d,k} \leq K_{d,max} \tag{45}$$
$$\lambda_{min} \leq \lambda_k \leq \lambda_{max} \tag{46}$$
$$\mu_{min} \leq \mu_k \leq \mu_{max} \tag{47}$$

where $e_k(t)$ denotes the instantaneous tracking error for the kth state, and $K_{p,min}$, $K_{i,min}$, $K_{d,min}$, λ_{min}, μ_{min} and $K_{p,max}$, $K_{i,max}$, $K_{d,max}$, λ_{max}, μ_{max} represent the lower and upper permissible boundaries for the design variables, respectively. These boundary limits are explicitly predefined based on the physical actuator saturation constraints of the quadrotor.

A major limitation of standard metaheuristic applications in control engineering is the blind minimization of error without guaranteeing mathematical system stability. While Theorem 1 analytically establishes the Lyapunov stability condition as $\dot{V}_k \leq 0$, it does not by itself guarantee that the parameter set produced by a metaheuristic search will satisfy this condition. Therefore, the derived stability condition ($\dot{V}_k \leq 0$) is directly embedded into the GTOA objective function as a hard penalty constraint, ensuring that the optimizer is mathematically bound to search exclusively within the stabilizing subset $\mathbb{S}_{stable}$ identified by Theorem 1. If a candidate parameter set Φ_k fails to guarantee a strictly negative derivative for the Lyapunov candidate function, a severe mathematical penalty factor is applied. This active constraint rejects unstable agents, steering the GTOA strictly toward the energy-dissipating regions of the tuning space. Consequently, the Lyapunov-constrained objective function utilized by the GTOA is represented as:

$$J_k(\Phi_k) = \begin{cases} \int_0^\infty t|e_k(t)|dt, & \text{if } \Phi_k \text{ ensures } \dot{V}_k \leq 0 \\ \infty, & \text{otherwise} \end{cases} \tag{48}$$

The mathematical implications of this active constraint are formalized in the following corollary:

Corollary 1. *(Algorithmic Constraint Satisfaction). Given the Lyapunov stability condition established in Theorem 1, if the GTOA algorithm minimizes the penalized objective function defined in Eq. (48), the resulting optimal parameter set $\Phi_k^* = \arg\min J_k(\Phi_k)$ is mathematically guaranteed to belong to the stabilizing set of the controller, ensuring the global asymptotic stability of the physical tracking error dynamics.*

Proof. Let $\mathbb{S}$ denote the feasible set of controller parameters bounded by the inequality constraints (Eqs. (43)–(47)). Let $\mathbb{S}_{stable} \subset \mathbb{S}$ define the stabilizing subset where the fractional energy dissipation condition $\dot{V}_k \leq 0$ holds true. According to the

piecewise formulation in Eq. (48), for any candidate vector $\Phi_k \notin \mathbb{S}_{stable}$, the cost function $J_k(\Phi_k) \to \infty$. Because the GTOA is mathematically designed to search for the global minimum, the infinite penalty strictly rejects unstable agents. Therefore, provided that a feasible solution exists within $\mathbb{S}_{stable}$, the minimum of the objective function must reside within the stable subset. Thus, $\Phi_k^* \in \mathbb{S}_{stable}$, thereby satisfying Lyapunov's direct method. □

Remark 2. *As mathematically implied by the Lyapunov stability proof, bounding the controller gains ($K_{p,min}, K_{i,min}$, $K_{d,min}$) to strictly positive values, and restricting the fractional orders within the bounded interval $(0,2)$, has a dual purpose. First, they define the feasible set $\mathbb{S}$ within which the Lyapunov stability condition established in Theorem 1 is structurally enforceable. Second, combined with the dynamic Lyapunov penalty mechanism in Eq. (48), they guarantee that the GTOA search is strictly confined to the stabilizing subset $\mathbb{S}_{stable} \subset \mathbb{S}$, ensuring that the physical control inputs derived in Lemma 1 remain within the operational limits of the hardware architecture.*

To sum up, the stability framework presented in this work operates through a unified two-layer architecture. At the *analytical layer*, Theorem 1 derives the Lyapunov stability condition and establishes the mathematical requirement that $\dot{V}_k \leq 0$ must hold to guarantee asymptotic convergence of the tracking errors. At the *computational layer*, the dynamic Lyapunov penalty mechanism in Eq. (48), combined with the search bound constraints established in Remark 2, enforces this analytical condition directly within the GTOA optimization process, ensuring that every evaluated candidate solution belongs to the stabilizing subset $\mathbb{S}_{stable}$. Corollary 1 then formally bridges these two layers by proving that the optimal parameter set Φ_k^* returned by the GTOA is mathematically guaranteed to satisfy the Lyapunov condition, with the non-emptiness of $\mathbb{S}_{stable}$. Consequently, the global asymptotic stability of the closed-loop quadrotor system is guaranteed not only theoretically through Lyapunov analysis, but also computationally enforced throughout the entire parameter search process.

3.4 Gorilla Troop Optimization Algorithm (GTOA)

The GTOA is a robust, gradient-free metaheuristic algorithm selected for its higher ability of avoiding premature convergence in highly non-convex spaces. It is formulated based on the behavioral characteristics of gorilla troops, where a dominant silverback governs decision-making, protects the group, and manages access to resources[33]. This is mirrored in GTOA, where the best-performing candidate solution acts as the "silverback" that guides the search. Troop members also display collective behaviors, as they adjust their movements based on both the environmental cues and the leader's actions; similarly, candidate solutions adapt during the optimization process. Additionally, gorillas assume different roles, with some individuals exploring unknown regions while others exploit resource-rich areas. This behavior enhances the adaptability and the robustness, and in the algorithm, it ensures a balanced between exploration and exploitation, leads to an efficient search process[43].

The computational execution of the GTOA is partitioned into two distinct mathematical phases: global exploration and local exploitation. The overall optimization procedure is illustrated in Figure 3, while the following steps highlight the execution of the GTOA optimizer:

Step 1. *Construction of the design variables vector Φ_k*: For each decoupled controller k, the vector of design variables is formally defined as:

$$\Phi_k = [K_{p,k}, K_{i,k}, K_{d,k}, \lambda_k, \mu_k]^T,\ \forall k \in \{x, y, z, \phi, \theta, \psi\} \tag{49}$$

Step 2. *Initialization*:

- Choose the population size N_{pop}.
- Choose the maximum number of iterations i_{max}.
- Choose the migration parameter p (usually set to 0.03[43]).
- Choose the exploitation parameter W (usually set to 0.8[43]).
- Set the iteration counter i to 1.

Step 3. *Initial generation of random population*: Generate a random population of candidate solutions (gorillas) as follows:

$$\Phi^0_{(k,j,n)} = \Phi_j^{min} + r_j \times (\Phi_j^{max} - \Phi_j^{min}), n \in \{1, 2, \dots, N_{pop}\}, j \in \{1, 2, \dots, \hat{D}\}, \quad k \in \{x, y, z, \phi, \theta, \psi\} \tag{50}$$

where $r_j \in [0,1]$ is a uniformly distributed random number, Φ_j^{max} and Φ_j^{min} represent the upper and lower boundaries of each design variable j, respectively, and $\hat{D}$ denotes the length of Φ_k. Here, $\hat{D} = 5$ represents the total number of tuning variables $(K_p, K_i, K_d, \lambda, \mu)$ for the kth FOPID controller.

To ensure algorithmic progression and prevent a scenario where all initially generated individuals violate the Lyapunov stability constraint (yielding infinite fitness penalties), an initial boundary constraint is applied. For each design variable j, the initial position is enforced as:

$$\Phi^0_{k,j,n} = \begin{cases} \Phi^{max}_j, & \text{if } \Phi^0_{k,j,n} > \Phi^{max}_j \\ \Phi^{min}_j, & \text{if } \Phi^0_{k,j,n} < \Phi^{min}_j \\ \Phi^0_{k,j,n}, & \text{otherwise} \end{cases} \tag{51}$$

Step 4. *Exploration phase*: In this phase, the algorithm widely samples the fractional parameter space to locate stabilizing regions.

Step 4.1 *Migration mechanism selection*: At the current iteration i, the candidate position vector $G\Phi^i_{k,n}$ for the nth gorilla is generated using one of three distinct mechanisms: migration to an unknown location, movement toward other gorillas, or

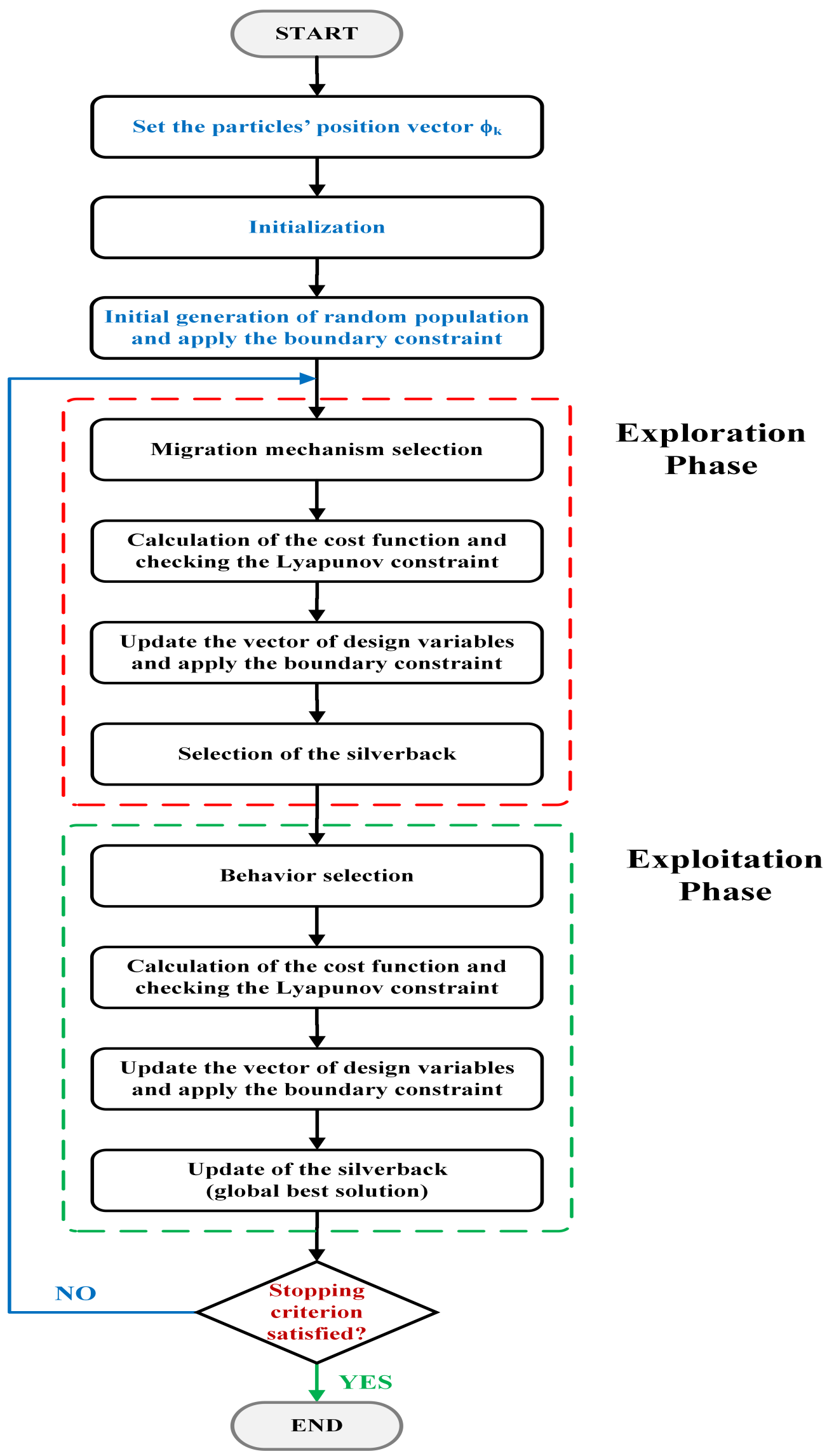

Figure 3. Flowchart of the proposed GTOA optimization process for tuning the FOPID controller.

migration toward a known location. The position update is mathematically modeled as:

$$G\Phi^{i}_{k,n} = \begin{cases} \Phi^{min} + r_1 \times (\Phi^{max} - \Phi^{min}), & rand < p \\ (r_2 - C) \times \Phi^{i-1}_{k,r} + L \times H, & rand \geq 0.5 \\ \Phi^{i-1}_{k,n} - L \times \Big(L \times \big(\Phi^{i-1}_{k,n} - G\Phi^{i}_{k,r}\big) + r_3 \times \big(\Phi^{i-1}_{k,n} - G\Phi^{i}_{k,r}\big)\Big), & p \leq rand < 0.5 \end{cases} \tag{52}$$

where $rand \in [0,1]$ acts as a stochastic decision switch drawn from a uniform distribution at each iteration, while $r_1, r_2, r_3 \in [0,1]$ are uniformly distributed random multipliers. The first condition ($rand < p$) dictates the migration to an unknown location, the second condition ($rand \geq 0.5$) governs the movement toward other gorillas, and the last condition ($p \leq rand < 0.5$) indicates the migration toward a known location. $\Phi^{i-1}_{k,r}$ is the position of a randomly selected gorilla from the population, $G\Phi^{i}_{k,r}$ is a randomly selected candidate vector from the newly generated positions, $r \neq n$, and $\Phi^{i-1}_{k,n}$ is the position of the nth gorilla. The parameters C, L, and H are calculated as follows:

$$C = \big(\cos(2r_4) + 1\big) \times \left(1 - \frac{i}{i_{max}}\right) \tag{53}$$

$$L = C \times l^{\backslash} \tag{54}$$

$$H = Z \times \Phi^{i-1}_{k,n} \tag{55}$$

where $r_4 \in [0,1]$ is a uniform random number, $l^{\backslash} \in [-1,1]$ is a randomly distributed variable, and Z is a random scalar bounded in $[-C, C]$. It is worth noting that C is a decaying inertia weight, L simulates the leadership behavior of the silverback, and H is the manipulation vector[43].

Step 4.2 *Objective function calculation and Lyapunov constraint handling*: Calculate the objective function presented in Eq. (48), and check for the generated candidate vector for satisfying the Lyapunov constraint:

$$J_k(G\Phi^{i}_{k,n}) = \begin{cases} \int_0^\infty t|e_k(t)|dt, & \text{if } \dot{V}_k \leq 0 \\ \infty, & \text{if } \dot{V}_k > 0 \end{cases} \tag{56}$$

Step 4.3 *Update the design variables' values*: For each gorilla, its vector of design variables $\Phi^{i}_{k,n}$ is updated as follows:

$$\Phi^{i}_{k,n} = \begin{cases} G\Phi^{i}_{k,n}, & \text{if } J_k(G\Phi^{i}_{k,n}) < J_k(\Phi^{i-1}_{k,n}) \\ \Phi^{i-1}_{k,n}, & \text{otherwise} \end{cases} \tag{57}$$

Step 4.4 *Boundary constraint enforcement*: For each design variable j, enforce the vector of updated design variables $\Phi^{i}_{k,n}$ to be bounded within the limits of the jth design variable:

$$\Phi^{i}_{k,j,n} = \begin{cases} \Phi^{max}_{j}, & \text{if } \Phi^{i}_{k,j,n} > \Phi^{max}_{j} \\ \Phi^{min}_{j}, & \text{if } \Phi^{i}_{k,j,n} < \Phi^{min}_{j} \\ \Phi^{i}_{k,j,n}, & \text{otherwise} \end{cases} \tag{58}$$

Step 4.5 *Select the silverback (the best solution)*: In the current iteration i, determine the best gorilla in the troop with the lowest objective function $J_{k,min}$. With its associated values of design variables Φ^{*}_{k}, It is designated as the silverback.

Step 5. *The exploitation phase*: In this phase, the population has two behaviors; follow the silverback and fight for the adult females.

Step 5.1 *Behavior selection*: The candidate position vector $G\Phi^{i}_{k,n}$ is updated as follows:

$$G\Phi^{i}_{k,n} = \begin{cases} L \times M \times \big(\Phi^{i-1}_{k,n} - \Phi^{*}_{k}\big) + \Phi^{i-1}_{k,n}, & C \geq W \\ \Phi^{*}_{k} - \big(\Phi^{*}_{k} \times Q - \Phi^{i-1}_{k,n} \times Q\big) \times A, & C < W \end{cases} \tag{59}$$

where the first condition $C \geq W$ indicates that the population follows the silverback, and when $C < W$, the population competes

for adult females. The parameters M, Q, and A are calculated as follows:

$$M = \left(\left| \frac{1}{N_{pop}} \sum_{n=1}^{N_{pop}} \Phi_{k,n}^{i-1} \right|^{\hat{g}} \right)^{\frac{1}{\hat{g}}} \tag{60}$$

$$\hat{g} = 2^L \tag{61}$$

$$Q = 2 \times r_5 - 1 \tag{62}$$

$$A = \beta \times E \tag{63}$$

$$E = \begin{cases} N_1, & \text{if } rand \geq 0.5 \\ N_2, & \text{if } rand < 0.5 \end{cases} \tag{64}$$

where $rand, r_5 \in [0,1]$ are uniformly distributed random values, N_1, and N_2 are random values drawn from standard normal distributions[43].

Step 5.2 *Objective function calculation and Lyapunov constraint handling*: Re-calculate the objective function for each candidate solution $G\Phi_{k,n}^{i}$, and check the Lyapunov constraint based on Eq. (56).

Step 5.3 *Update the design variables' values*: Re-update the vector of design variables $\Phi_{k,n}^{i}$ for each gorilla based on Eq. (57).

Step 5.4 *Boundary constraint enforcement*: Re-enforce the values of $\Phi_{k,n}^{i}$ to be bounded based on Eq. (58).

Step 5.5 *Update the silverback*: If a gorilla has a lower objective value ($J_{k,min}$) than the current silverback, update Φ_k^* to this new gorilla. Otherwise, the current silverback remains the updated silverback. This will be the optimal solution.

Step 6. *Termination*: Check for convergence. If the termination criterion is met, terminate the algorithm. Otherwise, set $i = i + 1$ and go to Step 4.

Remark 3. *The termination criterion applied in this work is that the optimization algorithm is ended if the objective function does not change after 20 iterations.*

4 Hardware-in-the-Loop (HIL) Experimental Validation

This section details the real-time HIL validation of the proposed control framework utilizing the OPAL-RT OP4510 real-time target simulator. The primary objective of these experimental tests is to evaluate the controller performance under physical operational constraints, specifically real-time computational latency, signal quantization, and communication latency, that are typically unmodeled in standard simulations. This HIL validation was performed in the Electronics Research Institute (ERI), Cairo, Egypt.

To ensure a rigorous and fair comparison, the proposed GTOA-FOPID is evaluated against three controllers: AVOA-FOPID, PSO-FOPID, and a conventional PSO-tuned integer-order PID. The comparative evaluation framework is structured along two independent perspectives to provide a rigorous assessment of the proposed architecture. From a *control perspective*, the FOPID controllers are evaluated against a conventional integer-order PID controller, with both tuned by PSO under identical conditions, thereby isolating the contribution of the fractional-order operators independently of the optimization strategy. From an *optimization perspective*, three metaheuristic algorithms: GTOA, AVOA, and PSO are applied to the same FOPID structure, isolating the contribution of the optimizer independently of the controller. AVOA is selected as a modern nature-inspired algorithm of comparable architectural complexity to GTOA, while PSO serves as the established standard baseline in the metaheuristic literature. This controlled experimental design ensures that each reported performance gain is attributed to a specific component of the proposed framework.

4.1 Experimental Setup

The OP4510 is a compact real-time simulator from OPAL-RT Technologies widely used for Rapid Control Prototyping (RCP) and HIL applications. It features a powerful heterogeneous computational architecture combining a multi-core processor with a Xilinx Kintex-7 FPGA. As illustrated in Figure 4, the host PC serves as the command station; it compiles the MATLAB/Simulink models into executable C-code and deploys it to the OP4510 target hardware via a high-speed TCP/IP Ethernet connection. To interface with external hardware, the simulator employs 16-bit resolution Analog-to-Digital (ADC) and Digital-to-Analog (DAC) converters, allowing us to evaluate the controller's robustness against real-world signal quantization and sensor noise. To ensure a realistic evaluation, the real-time execution step size was strictly maintained at 10^{-4} s (10 kHz sampling frequency). The optimal controller gains (Φ^*) of the GTOA-FOPID were deployed directly onto the real-time processor. The resulting analog

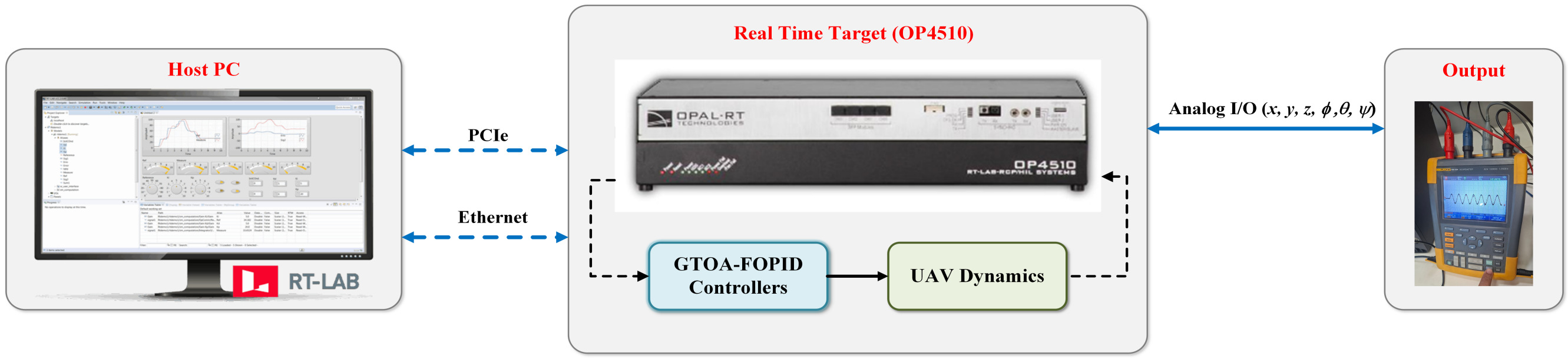

Figure 4. OPAL-RT OP4510 HIL experimental setup.

voltage outputs, representing the UAV's continuous dynamic response, were captured using an external digital oscilloscope for comparative analysis. Given the physical 4-channel display limitation of the external data acquisition oscilloscope, a hybrid visualization approach was adopted: representative axes were captured via raw analog oscilloscope traces to prove real-time execution, while complex trajectories and full state histories were exported to MATLAB for complete analysis. Furthermore, to facilitate real-time fractional operator computation on the OP4510, Oustaloup's recursive approximation is employed to discretize the fractional-order operators (λ_k, μ_k) within the deployed FOPID controllers, with a fifth-order approximation applied over the frequency band $[\omega_l, \omega_h]$ = $[10^{-3}, 10^{3}]$ rad/s. The physical parameters of the Autel EVO Max 4T UAV applied throughout this hardware execution[44] are detailed in Table 1.

4.2 Statistical Optimization Performance Analysis

To assess the statistical robustness of the optimization process, each of the four optimization algorithms is executed across 20 independent runs with randomized initial populations. The mean and standard deviation (STD) of the achieved ITAE, alongside the average number of iterations to convergence and the mean execution time, are summarized in Tables 2 and 3. Note that, the optimal gain set Φ_k^* achieving the best ITAE across all runs is subsequently deployed on the OPAL-RT OP4510 for real-time HIL validation, ensuring that the HIL results reflect the best achievable performance of each algorithm rather than a single stochastic run.

As depicted in Table 2, across all three representative axes, the GTOA-FOPID achieves the lowest average ITAE and standard deviation among all evaluated algorithms, demonstrating both superior optimality and consistent reproducibility over 20 independent runs. In the under-actuated x-axis, the GTOA-FOPID yields a mean ITAE of 1.309, representing reductions of 83.4%, 66.6%, and 50.0% compared to PSO-PID, PSO-FOPID, and AVOA-FOPID, respectively. Similarly in the altitude z-axis, where GTOA-FOPID achieves a mean ITAE of 13.02, corresponding to reductions of 71.6% and 59.7% over PSO-PID and PSO-FOPID, respectively. Furthermore, the STD of GTOA-FOPID remains the lowest across all axes, highlighting effectiveness of the GTOA's exploration-exploitation mechanism, and confirming of the dynamic Lyapunov-constrained search space effectively steers the algorithm consistently toward the global minimum.

Table 3 highlights the computational efficiency of the four algorithms. As noticed, the integer-order PSO-PID exhibited a lower average execution time (80.5 s) and an average of 32.2 iterations, owing to its reduced three-dimensional parameter space. However, this comes at the cost of the ITAE performance, as demonstrated above. Conversely, within the expanded five-dimensional fractional search space, GTOA-FOPID converges in an average of 82.2 iterations, representing reductions of 57.2% and 26.8% compared to PSO-FOPID (192.2 iterations) and AVOA-FOPID (112.3 iterations), respectively. It is emphasized that this execution time corresponds entirely to the offline tuning phase. Once the optimal gains Φ_k^* are identified and deployed, the FOPID control law itself operates at $\mathscr{O}(1)$ complexity per control cycle, confirming its suitability for real-time execution at the 10 kHz sampling frequency of the OPAL-RT OP4510 platform.

Following this rigorous statistical evaluation, the best-performing parameter set synthesized by the GTOA-FOPID framework across the 20 runs was selected for the HIL deployment. The specific search boundaries utilized during the optimization phase, alongside the final optimal fractional and integer gains extracted for all six decoupled control axes, are detailed in Table 4. These optimized parameters constitute the exact control laws deployed in the subsequent real-time HIL evaluation.

$I_{xx} = 0.03\ \mathrm{kg \cdot m^2}$	$I_{yy} = 0.03\mathrm{kg \cdot m^2}$	$I_{zz} = 0.04\ \mathrm{kg \cdot m^2}$
$m = 1.7\mathrm{kg}$	$L = 0.2\mathrm{m}$	$K_{yaw} = 4\mathrm{N \cdot m}$

Table 1. UAV Parameters

Axis	Algorithm	Mean	STD
x	GTOA-FOPID	**1.309**	**0.004**
	AVOA-FOPID	2.616	0.006
	PSO-FOPID	3.923	0.012
	PSO-PID	7.893	0.015
z	GTOA-FOPID	**13.020**	**0.073**
	AVOA-FOPID	16.870	0.090
	PSO-FOPID	32.285	0.240
	PSO-PID	45.800	0.401
ψ	GTOA-FOPID	**0.359**	**0.004**
	AVOA-FOPID	0.519	0.007
	PSO-FOPID	0.923	0.013
	PSO-PID	0.951	0.017

Table 2. Statistical analysis of ITAE over 20 independent runs for axes x, z, and ψ.

Algorithm	Avg. Iterations	Avg. Exec. Time (s)
GTOA-FOPID	82.2	97.26
AVOA-FOPID	112.3	101.76
PSO-FOPID	192.2	107.84
PSO-PID	**32.3**	**80.46**

Table 3. Computational efficiency of the optimization algorithms over 20 independent runs.

4.3 Real-Time Trajectory Tracking Evaluation

Figure 5 shows the real-time HIL step response for the altitude (z-axis) control, where the quadrotor is commanded to reach a target height of 10 m. To provide a comprehensive multi-algorithm comparison utilizing the full capacity of the oscilloscope, the responses are mapped to four distinct channels: the proposed GTOA-FOPID is displayed on Channel A (red), AVOA-FOPID on Channel B (blue), PSO-FOPID on Channel C (black), and the baseline PSO-PID on Channel D (green). As observed in the standard transient view in Figure 5(a), the proposed GTOA-FOPID achieves the highest rise time, rapidly overcoming

Controller Axis	K_p	K_i	K_d	λ	μ
Bounds (z)	[0.1, 25]	[0, 10]	[0, 10]	[0.1, 1.5]	[0.1, 1.5]
Bounds (x, y)	[0.1, 10]	[0, 5]	[0, 5]	[0.1, 1.5]	[0.1, 1.5]
Bounds (ϕ, θ, ψ)	[0.1, 20]	[0, 8]	[0, 8]	[0.1, 1.5]	[0.1, 1.5]
Altitude (z)	12.45	5.12	3.85	0.852	0.915
Longitudinal (x)	2.45	0.85	1.12	0.91	0.85
Lateral (y)	2.58	0.89	1.08	0.935	0.83
Roll (ϕ)	8.42	2.15	1.85	0.895	0.912
Pitch (θ)	8.55	2.21	1.82	0.91	0.898
Yaw (ψ)	4.12	1.05	0.82	0.825	0.85
N_{pop}	30				
i_{max}	100				

Table 4. Algorithmic parameters, search bounds and optimal GTOA-FOPID controller parameters for the quadrotor UAV.

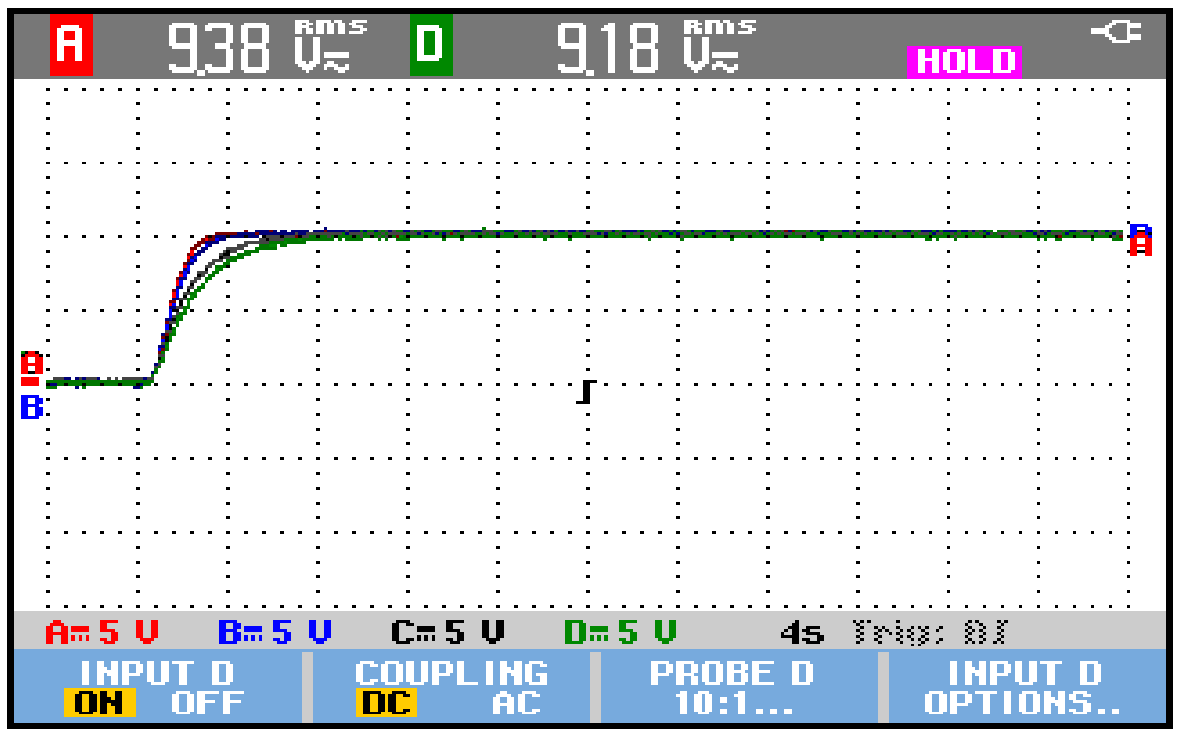

(a) Normal transient view

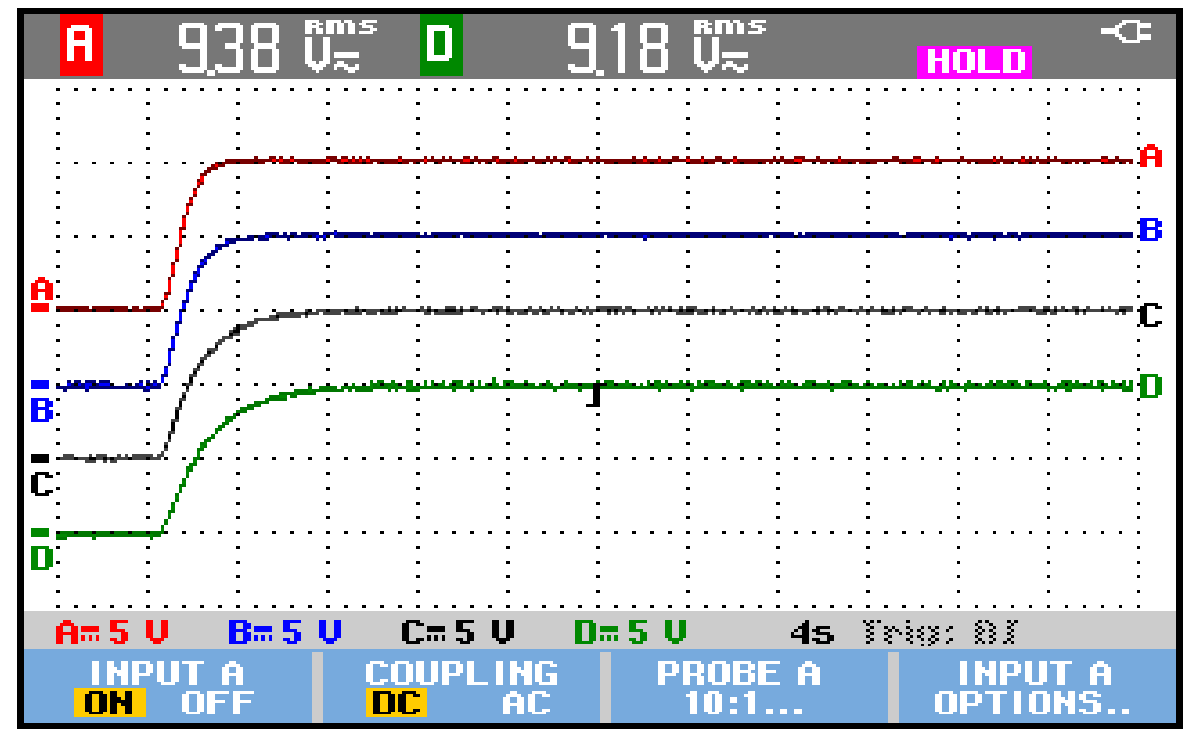

(b) Zoomed steady-state view (vertically separated for clarity)

Figure 5. Real-time HIL altitude (z-axis) step response targeting a 10 m reference. The GTOA-FOPID (Red) is compared against AVOA-FOPID (Blue), PSO-FOPID (Black), and PSO-PID (Green).

gravitational forces to reach the 10 m reference significantly faster than the other algorithms. To allow for a precise examination of the steady-state convergence without trace overlap, the hardware signals were vertically separated in the zoomed view presented in Figure 5(b). This magnified perspective physically validates that the GTOA-FOPID exhibits a smooth settling phase compared to the other algorithms.

Figure 6 illustrates the real-time execution of tracking a continuous circular trajectory at an altitude of 10 m. As observed, the baseline PSO-PID exhibits massive deviations, significant lag, and struggles to maintain altitude, particularly during the initial lift and the transition into planar circular motion. Conversely, the GTOA-FOPID maintains consistent and precise adherence to the reference trajectory throughout the entire flight. To investigate the underlying transient behavior driving this scenario, the raw hardware signals for the highly coupled x-axis were captured.

Figure 7 compares the step response of the proposed GTOA-FOPID against the baseline PSO-PID controller. The full transient view in Figure 7(a) confirms the convergence of the under-actuated FOPID controllers. However, the magnified steady-state view in Figure 7(b) physically verifies that the GTOA-FOPID (blue) successfully converges to the desired trajectory x_r (red) better than the PSO-PID (green). This can be highlighted in Figure 8, as results clearly demonstrate that FOPID architecture outperforms the PSO-PID especially in the highly coupled under–actuated x and y axes. It can be noticed that

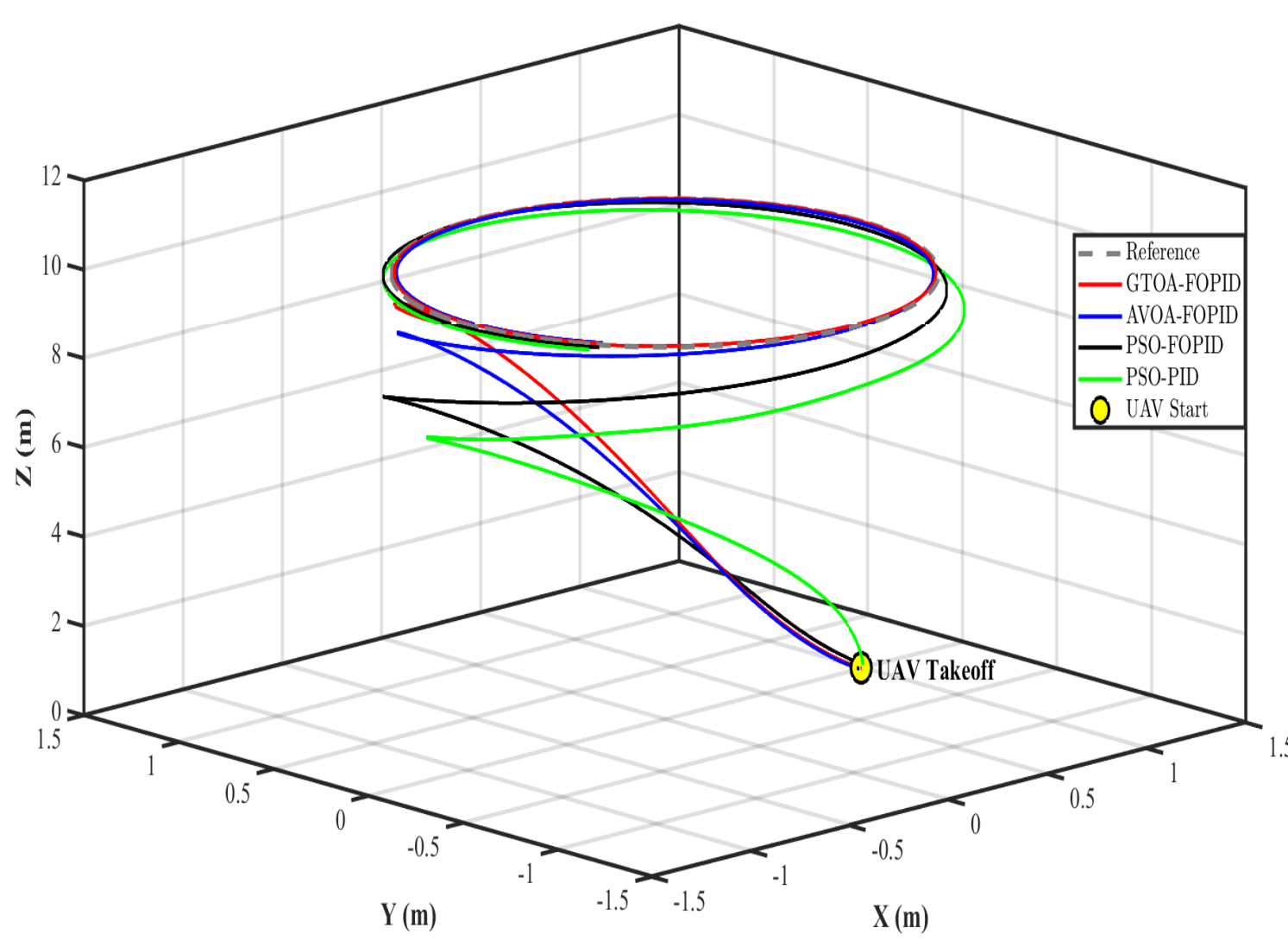

Figure 6. Real-time HIL 3D trajectory tracking.

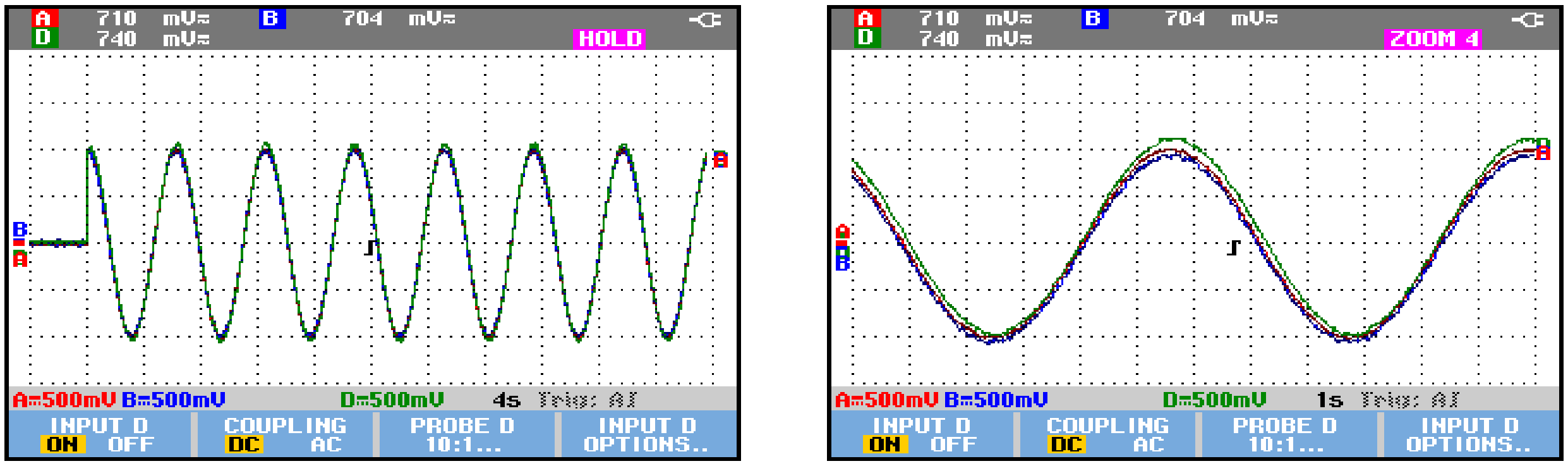

(a) Normal transient view (b) Zoomed steady-state view

Figure 7. Real-time HIL step response for the x-axis. The proposed GTOA-FOPID (blue) is compared against PSO-PID (Green), and the desired trajectory x_r (red)

PSO-PID yields massive initial transient errors (exceeding 2 m to 4 m in e_x and e_y) and persistent steady-state oscillations. Furthermore, when evaluating the metaheuristic optimization strategies, it is clear that GTOA-FOPID achieves better results than both AVOA-FOPID and PSO-FOPID, achieving lowest steady-state error and shortest settling time, validating its performance under real-world hardware constraints.

Table 5 details the transient response control metrics for the altitude z and yaw ψ. As can be noticed, FOPID outperforms the conventional PID in all metrics. For the altitude tracking, GTOA-FOPID demonstrated marked performance, achieving a rapid rise time of approximately 1.72 s, and reducing the settling time to just 2.96 s. This is effectively less than the convergence time of the baseline PSO-PID by more than half, while maintaining a practically negligible overshoot of 0.16%. In the yaw axis, while the PSO-FOPID achieves a marginally faster rise time (0.91 s), it exhibits nearly 7% overshoot. Conversely, the GTOA-FOPID gets the optimal control balance, limiting the overshoot to about 0.78% while settling rapidly at 1.86 s. Furthermore, across both axes, the FOPID algorithms exhibit exceptional steady-state error (e_{ss} bounded between 10^{-2} and 10^{-3}, proving that the fractional integral operator successfully neutralizes the unmodeled aerodynamic disturbances that degrade

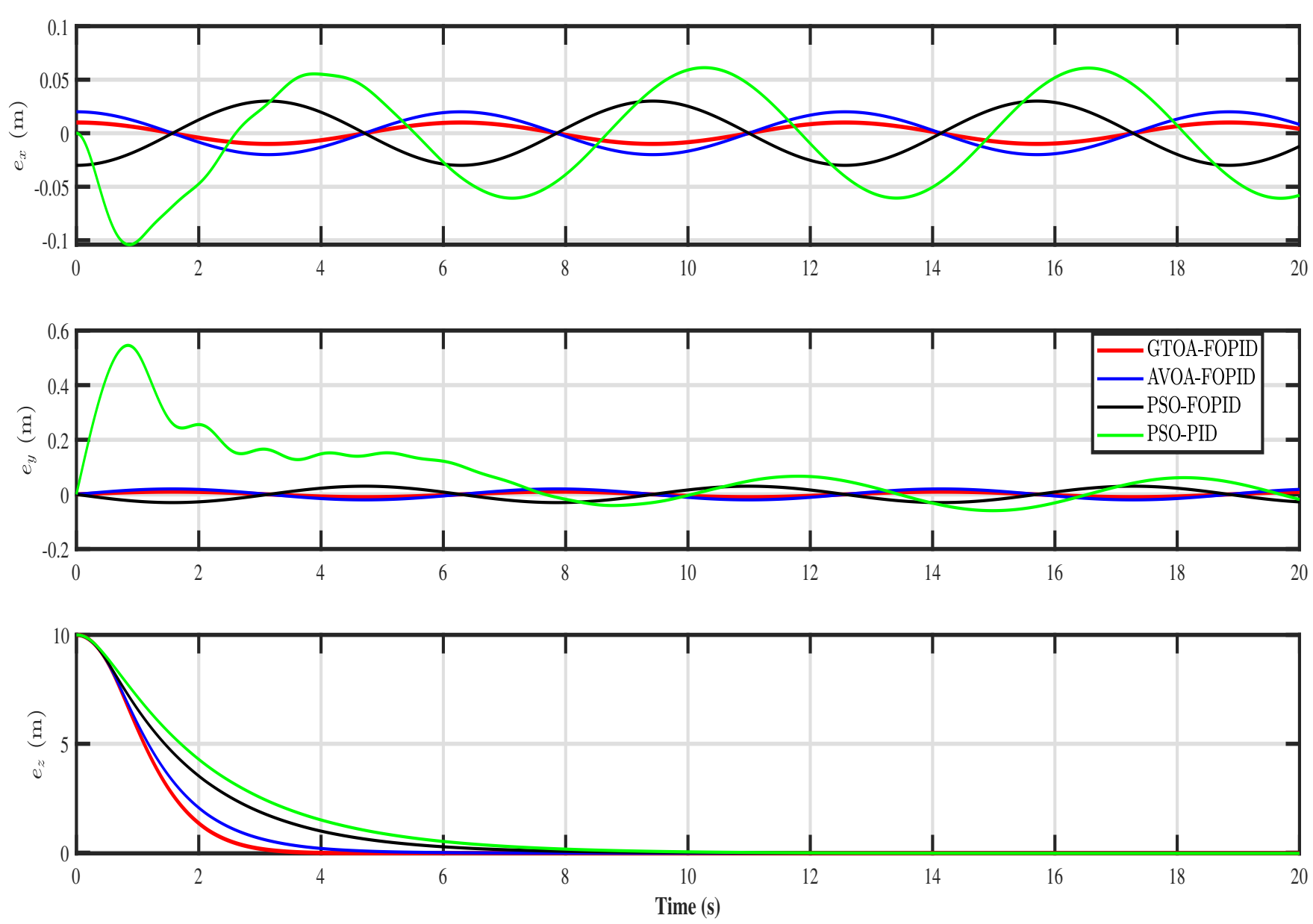

Figure 8. Real-time translational error (e_x, e_y, e_z).

State	Algorithm	t_d (s)	t_r (s)	t_s (s)	OS (%)	e_{ss}
z	GTOA-FOPID	**1.1076**	**1.7200**	**2.9640**	**0.16**	$\mathbf{1.63 \times 10^{-2}}$
	AVOA-FOPID	1.1748	2.1858	3.9814	**0.16**	$\mathbf{1.63 \times 10^{-2}}$
	PSO-FOPID	1.4440	3.5350	6.4603	**0.16**	$\mathbf{1.63 \times 10^{-2}}$
	PSO-PID	1.7059	4.2825	7.6825	0.31	3.06×10^{-2}
ψ	GTOA-FOPID	**0.5642**	1.3617	**1.8578**	**0.78**	$\mathbf{1.58 \times 10^{-3}}$
	AVOA-FOPID	1.6469	1.6298	5.3250	2.84	$\mathbf{1.58 \times 10^{-3}}$
	PSO-FOPID	1.1092	**0.9131**	3.8499	6.98	1.79×10^{-3}
	PSO-PID	1.3926	3.2663	5.3871	0.93	2.03×10^{-3}

Table 5. Transient response analysis for altitude (z) and yaw (ψ). (t_d: Delay Time, t_r: Rise Time, t_s: Settling Time, OS: Overshoot, e_{ss}: Steady-State Error).

standard integer-order PID controllers.

Table 6 summarizes the trajectory tracking performance across four distinct error metrics. To provide a comprehensive evaluation without redundancy, three axes are selected; the highly-coupled and under-actuated x-axis, the altitude and yaw axes z, and ψ, respectively. While ITAE is considered in the optimization problem, results shows that GTOA-FOPID yields exceptional performance across all metrics. For instance, in the x-axis, the GTOA-FOPID not only minimizes the ITAE to 1.308, which is about 83% reduction compared to the baseline PSO-PID, but it also minimizes the mean square error (MSE) and the integral time square error (ITSE), confirming the efficiency of the FOPID fractional parameters compared to the integral order PID, and the superiority of the GTOA compared to both AVOA and PSO.

To validate the Lyapunov stability established in Theorem 1, the phase portraits of the tracking error dynamics (e vs. $\dot{e}$) of for both z and x axes are presented in Figure 9. As depicted in the z-axis portrait, the proposed GTOA-FOPID exhibits a superior performance over the other techniques in response to the 10 m altitude command. It achieves a peak error velocity ($\approx$ 6.6 m/s) for rapid descent before converging smoothly to the origin. On the other hand, the base line PSO-PID achieves the slowest response to reach the target attractor. In the x-axis portrait, the baseline PSO-PID controller exhibit a wider, less organized convergence trajectory with noticeable cycle oscillations near the origin $(0,0)$. This behavior is a direct indicator of underdamped transient behavior and poor kinetic energy dissipation. Conversely, the optimally tuned fractional operators within FOPID architectures dictate a significantly tighter and more direct convergence toward the equilibrium point. However,

State	Algorithm	IAE	MSE	ITAE	ITSE
**X-Axis (x)**	GTOA-FOPID	**0.010**	$\mathbf{5.09 \times 10^{-5}}$	**1.308**	$\mathbf{1.04 \times 10^{-2}}$
	AVOA-FOPID	0.020	2.04×10^{-4}	2.615	4.14×10^{-2}
	PSO-FOPID	0.030	4.58×10^{-4}	3.923	9.32×10^{-2}
	PSO-PID	0.104	2.16×10^{-3}	7.893	3.84×10^{-1}
**Altitude (z)**	GTOA-FOPID	**12.53**	**4.25**	**13.02**	**46.37**
	AVOA-FOPID	14.16	4.53	16.87	55.48
	PSO-FOPID	19.15	5.52	32.26	94.66
	PSO-PID	22.66	6.44	45.75	132.26
**Yaw (ψ)**	GTOA-FOPID	**0.229**	$\mathbf{1.81 \times 10^{-3}}$	**0.359**	$\mathbf{1.27 \times 10^{-2}}$
	AVOA-FOPID	0.384	4.23×10^{-3}	0.512	4.41×10^{-2}
	PSO-FOPID	0.564	6.18×10^{-3}	0.922	9.56×10^{-2}
	PSO-PID	0.542	4.67×10^{-3}	0.951	7.56×10^{-2}

Table 6. Trajectory Tracking Error Metrics for Representative Translational (x,z) and Rotational (ψ) Axes. (IAE: Integral of Absolute Error, MSE: Mean Squared Error, ITAE: Integral of Time-weighted Absolute Error, ITSE: Integral of Time-weighted Squared Error).

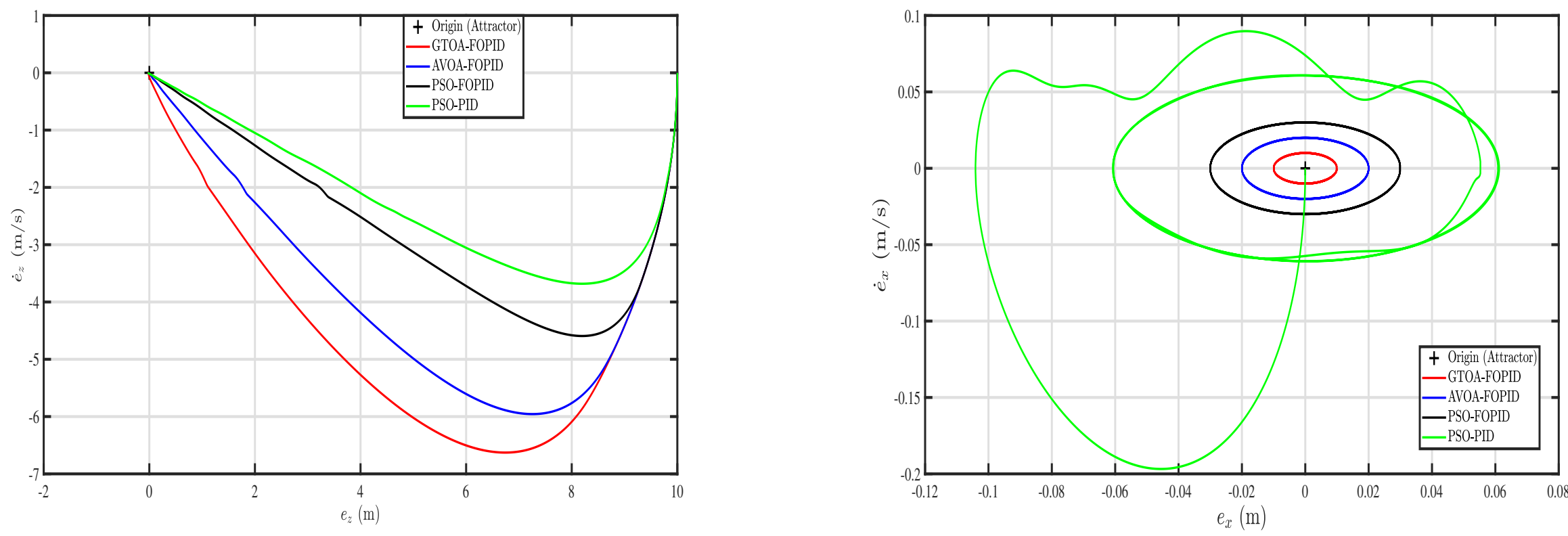

(a) z-axis (b) x-axis

Figure 9. Real-time phase portraits for the z and x axes

the GTOA-FOPID strictly outperforms the AVOA and PSO. This smooth phase trajectory confirms the global asymptotic stability of the system and validates the architecture's robustness under physical real-time constraints.

5 Conclusion

This paper presents a robust FOPID control architecture for the precise 6-DoF trajectory tracking of quadrotor UAVs, optimally tuned via the GTOA and evaluated against both the AVOA and PSO for comprehensive comparative evaluation. A two-layer stability framework is established: at the analytical layer, Lyapunov theory is employed to mathematically derive the asymptotic stability condition for the decoupled UAV subsystems; at the computational layer, a dynamic Lyapunov penalty mechanism is embedded within the GTOA objective function to enforce this condition across all candidate solutions throughout the optimization process, thereby guaranteeing the global asymptotic stability of the closed-loop system. The control performance is validated through real-time HIL experiments conducted on an industrial OPAL-RT OP4510 real-time target simulator, demonstrating that the theoretical stability guarantees translate into reliable performance under physical hardware constraints, including signal quantization, computational latency, and communication delays. Several notable features are demonstrated by the proposed scheme: (1) it achieves exceptional tracking precision and fast transient responses under the hardware constraints of the HIL platform, where the iso-damping properties of the fractional-order operators provide robustness against signal quantization and real-time computational delays; (2) it is computationally efficient and can be implemented in real-time, as the HIL experiments confirm that the theoretical stability results translate into reliable performance on actual hardware; and (3) while both modern algorithms significantly outperform traditional PSO-PID and PSO-FOPID baselines, GTOA achieves superior convergence and robustness, yielding the lowest ITAE, fastest settling time, and minimal steady-state error across all evaluated axes.

Acknowledgements

The authors would like to acknowledge the Electronics Research Institute (ERI), Egypt, for providing the laboratory facilities and the OPAL-RT 4510 real-time simulator used to conduct the Hardware-in-the-Loop (HIL) experimental validation in this study.

Funding

No funding has been received for this article.

Data Availability

All HIL data are included in this article.

Author contributions statement

B.E. contributed to conceptual design, methodology, validation, and original draft preparation. M.M.E. contributed to methodology and real-time validation. E.S contributed to supervision and discussion. I.E. contributed to supervision, correction of the manuscript, and discussion. M.A.K contributed to supervision, methodology, validation, thoroughly correction of the manuscript, and final draft preparation.

Competing interests

The authors declared that there are no conflicts of interest to declare that are relevant to this study.

Declarations

Ethics approval and consent to participate: The authors confirm that there are no potential acts of misconduct in this work, and approve of the journal upholding the integrity of the scientific record.

Additional information

The corresponding author is responsible for submitting a competing interests statement on behalf of all authors of the paper.

References

1. Kamel, M. A., Yu, X. & Zhang, Y. Formation control and coordination of multiple unmanned ground vehicles in normal and faulty situations: A review. *Annu. Rev. Control.* **49**, 128–144, DOI: 10.1016/j.arcontrol.2020.02.001 (2020).
2. Xiong, J.-J. & Zhang, G.-B. Global fast dynamic terminal sliding mode control for a quadrotor UAV. *ISA Transactions* **66**, 233–240, DOI: 10.1016/j.isatra.2016.09.019 (2017).
3. Wang, Z., Zou, Y., Liu, Y. & Meng, Z. Distributed control algorithm for leader-follower formation tracking of multiple quadrotors: Theory and experiment. *IEEE/ASME Transactions on Mechatronics* **26**, 1095–1105, DOI: 10.1109/tmech.2020.3017816 (2021).
4. Cowling, I. D., Yakimenko, O. A., Whidborne, J. F. & Cooke, A. K. A prototype of an autonomous controller for a quadrotor uav. In *European Control Conference (ECC)*, 4001–4008, DOI: 10.23919/ECC.2007.7068316 (2007).
5. Durdevic, P., Ortiz-Arroyo, D., Li, S. & Yang, Z. Vision aided navigation of a quad-rotor for autonomous wind-farm inspection. *IFAC-PapersOnLine* **52**, 61–66, DOI: 10.1016/j.ifacol.2019.08.049 (2019).
6. Dydek, Z. T., Annaswamy, A. M. & Lavretsky, E. Adaptive control of quadrotor UAVs: A design trade study with flight evaluations. *IEEE Transactions on Control. Syst. Technol.* **21**, 1400–1406, DOI: 10.1109/TCST.2012.2200104 (2013).
7. Dierks, T. & Jagannathan, S. Output feedback control of a quadrotor uav using neural networks. *IEEE Transactions on Neural Networks* **21**, 50–66, DOI: 10.1109/TNN.2009.2034145 (2010).
8. Lopez-Sanchez, I. & Moreno-Valenzuela, J. PID control of quadrotor uavs: A survey. *Annu. Rev. Control.* **56**, 1–21, DOI: https://doi.org/10.1016/j.arcontrol.2023.100900 (2023).
9. Suhail, S. A., Bazaz, M. A. & Hussain, S. Altitude and attitude control of a quadcopter using linear active disturbance rejection control. In *International Conference on Computing, Power and Communication Technologies (GUCON)*, 281–286 (2019).
10. Madani, T. & Benallegue, A. Sliding mode observer and backstepping control for a quadrotor unmanned aerial vehicles. In *American Control Conference*, 5887–5892, DOI: 10.1109/ACC.2007.4282548 (2007).
11. Ghamry, K. A. & Zhang, Y. Formation control of multiple quadrotors based on leader-follower method. In *International Conference on Unmanned Aircraft Systems (ICUAS)*, 1037–1042, DOI: 10.1109/icuas.2015.7152394 (2015).
12. Al-Mahturi, A., Santoso, F., Garratt, M. A. & Anavatti, S. G. *Modeling and control of a quadrotor unmanned aerial vehicle using type-2 fuzzy systems*, 25–46 (Elsevier, 2021).
13. Teng, Y.-F., Hu, B., Liu, Z.-W., Huang, J. & Guan, Z.-H. Adaptive neural network control for quadrotor unmanned aerial vehicles. In *Asian Control Conference (ASCC)*, 988–992, DOI: 10.1109/ascc.2017.8287305 (2017).
14. Wen, G., Hao, W., Feng, W. & Gao, K. Optimized backstepping tracking control using reinforcement learning for quadrotor unmanned aerial vehicle system. *IEEE Transactions on Syst. Man, Cybern. Syst.* **52**, 5004–5015, DOI: 10.1109/tsmc.2021.3112688 (2022).

15. Aliyari, M. *et al.* Design and implementation of a constrained model predictive control approach for unmanned aerial vehicles. *IEEE Access* **10**, 91750–91762, DOI: 10.1109/ACCESS.2022.3202020 (2022).

16. Wu, H., Bai, J. & Zou, H. Fast predictive functional control based on auxiliary variable optimization. *The Can. J. Chem. Eng.* DOI: https://doi.org/10.1002/cjce.70455.

17. Chen, Y., Hu, C., Bai, J. & Zou, H. Improved dynamic matrix control using co-optimization of dynamic control and economic performance enhancement. *The Can. J. Chem. Eng.* **104**, 4353–4367, DOI: https://doi.org/10.1002/cjce.70295 (2026).

18. Aissa, B., Med Amine, H. & Imade Eddine, K. Metaheuristic optimization of fractional-order PID controllers for quadcopter system. In *International Conference on Embedded Distributed Systems (EDiS)*, 314–319, DOI: 10.1109/edis63605.2024.10783289 (2024).

19. Zaki, H., Rashid, A. & Masud, U. Lyapunov-based fractional-order PID controller design for coupled nonlinear system. *Transactions Inst. Meas. Control.* **47**, 1046–1056, DOI: 10.1177/01423312241262067 (2025).

20. Elbasueny, B., Kamel, M. A., Said, E. & Elsherif, I. A. Cooperative control of multiple UAVs based on optimized fractional–order PID controller. In *International Conference on Electrical Engineering (ICEENG)*, 1–6, DOI: 10.1109/ICEENG64546.2025.11031285 (2025).

21. Saha, S. *et al.* Fractional order phase shaper design with bode's integral for iso-damped control system. *ISA Transactions* **49**, 196–206, DOI: https://doi.org/10.1016/j.isatra.2009.12.001 (2010).

22. Ali Mahmood, A. A., Mohammed Almaged. Design of fractional order PID controller based on genetic algorithm optimization for vertical take-off and landing platforms. *J. Optim. Decis. Mak.* **2**, 263–269 (2023).

23. Wendimu, A. A., Matušů, R., Shaikh, I. & Kebede, Z. K. Fractional-order PID control for elevation and azimuth in a twin rotor system. *Sci. Reports* **15**, 1–30, DOI: 10.1038/s41598-025-18763-8 (2025).

24. Espinoza, J. *et al.* Fractional-order PID control of quadrotor drone. In *Innovations in Power and Advanced Computing Technologies (i-PACT)*, 1–6, DOI: 10.1109/i-pact58649.2023.10434503 (2023).

25. Shao, K., Liu, J. & Li, M. Fractional order pid control of quadrotor UAV based on SA-PSO algorithm. In *Third International Conference on Computer Science and Communication Technology (ICCSCT 2022)*, DOI: 10.1117/12.2662180 (2022).

26. Rosmadi, N. H. B. *et al.* Fractional-order control algorithm for tello EDU quadrotor drone safe landing during disturbance on propeller. *Drones* **8**, 566, DOI: 10.3390/drones8100566 (2024).

27. Benhamou, A., Hartani, M. A., Kimi, I. E., Soumeur, M. A. & Baba, M. Bio-inspired optimization of fractional-order control in drone systems. *J. Elecetrical Eng. Renew. Energies Dev.* **1**, 26–32 (2025).

28. Ziani, F., Benhammou, A., Hartani, M. A. & Rezk, H. Quadrotor robust fractional-order control based on a recent bonobo optimization algorithm. *J. Math.* **2025**, 1–19, DOI: 10.1155/jom/2065604 (2025).

29. Saribas, H. & Kahvecioglu, S. PSO and GA tuned conventional and fractional order PID controllers for quadrotor control. *Aircr. Eng. Aerosp. Technol.* **93**, 1243–1253, DOI: 10.1108/aeat-08-2020-0185 (2021).

30. Bian, L., Sun, W. & Sun, T. Trajectory following and improved differential evolution solution for rapid forming of uav formation. *IEEE Access* **7**, 169599–169613, DOI: 10.1109/ACCESS.2019.2954408 (2019).

31. Lin, S., Li, F., Li, X., Jia, K. & Zhang, X. Improved artificial bee colony algorithm based on multi-strategy synthesis for UAV path planning. *IEEE Access* **10**, 119269–119282, DOI: 10.1109/ACCESS.2022.3218685 (2022).

32. Yu, X., Jiang, N., Wang, X. & Li, M. A hybrid algorithm based on grey wolf optimizer and differential evolution for UAV path planning. *Expert. Syst. with Appl.* **215**, DOI: https://doi.org/10.1016/j.eswa.2022.119327 (2023).

33. You, J. *et al.* Modified artificial gorilla troop optimization algorithm for solving constrained engineering optimization problems. *Mathematics* **11**, DOI: 10.3390/math11051256 (2023).

34. Abdollahzadeh, B., Gharehchopogh, F. S. & Mirjalili, S. African vultures optimization algorithm: A new nature-inspired metaheuristic algorithm for global optimization problems. *Comput. Ind. Eng.* **158**, DOI: 10.1016/j.cie.2021.107408 (2021).

35. Elbasueny, B., Kamel, M. A., Said, E. & Elsherif, I. A. Metaheuristic optimization of fractional order PID controllers for quadrotor trajectory tracking. In *International Youth Conference on Radio Electronics, Electrical and Power Engineering (REEPE)*, 1–8, DOI: 10.1109/REEPE69046.2026.11481283 (2026).

36. Ryll, M., Bülthoff, H. H. & Giordano, P. R. A novel overactuated quadrotor unmanned aerial vehicle: Modeling, control, and experimental validation. *IEEE Transactions on Control. Syst. Technol.* **23**, 540–556, DOI: 10.1109/TCST.2014.2330999 (2015).

37. Mo, F., Zhang, Z. & Yu, L. A control system of quadrotor uav with dynamic model. *Procedia Comput. Sci.* **247**, 1170–1178, DOI: https://doi.org/10.1016/j.procs.2024.10.141 (2024).

38. Mofid, O. & Mobayen, S. Adaptive sliding mode control for finite-time stability of quad-rotor uavs with parametric uncertainties. *ISA Transactions* **72**, 1–14, DOI: 10.1016/j.isatra.2017.11.010 (2018).

39. Bouabdallah, S. & Siegwart, R. Backstepping and sliding-mode techniques applied to an indoor micro quadrotor. In *IEEE International Conference on Robotics and Automation (ICRA)*, 2247–2252, DOI: 10.1109/robot.2005.1570447 (2005).

40. Zhang, Y. M. *et al.* Development of advanced FDD and FTC techniques with application to an unmanned quadrotor helicopter testbed. *J. Frankl. Inst.* **350**, 2396–2422, DOI: http://dx.doi.org/10.1016/j.jfranklin.2013.01.009 (2013).

41. Kamel, M. A., Ghamry, K. A. & Zhang, Y. Real-time fault-tolerant cooperative control of multiple uavs-ugvs in the presence of actuator faults. *J. Intell. & Robotic Syst.* **88**, 469–480, DOI: 10.1007/s10846-016-0463-8 (2017).

42. Oliveira, D. S. & Capelas de Oliveira, E. On a Caputo-type fractional derivative. *Adv. Pure Appl. Math.* **10**, 81–91, DOI: 10.1515/apam-2017-0068 (2019).

43. Abdollahzadeh, B., Soleimanian Gharehchopogh, F. & Mirjalili, S. Artificial gorilla troops optimizer: A new nature-inspired metaheuristic algorithm for global optimization problems. *Int. J. Intell. Syst.* **36**, 5887–5958, DOI: 10.1002/int.22535 (2021).

44. Autel Robotics. *EVO Max 4T User Manual*. Autel Robotics Co., Ltd. (2023). Available at https://www.autelrobotics.com/.
